# Dynamics of atmospheric ^131^I in radioactive plumes in eastern Japan immediately after the Fukushima accident by analysing published data

**DOI:** 10.1038/s41598-019-49379-4

**Published:** 2019-09-13

**Authors:** Haruo Tsuruta, Yuichi Moriguchi, Teruyuki Nakajima

**Affiliations:** 1 Remote Sensing Technology Center of Japan, Minato-ku, Tokyo, 105-0001 Japan; 2 Graduate School of Engineering, The University of Tokyo, Bunkyo-ku, Tokyo, 113-8656 Japan; 3 Earth Observation Research Center, Japan Aerospace Exploration Agency, Sengen, Tsukuba, 305-8505 Japan

**Keywords:** Environmental chemistry, Environmental chemistry

## Abstract

The spatio-temporal distribution of atmospheric radioiodine immediately after the Fukushima Daiichi Nuclear Power Plant (FD1NPP) accident has not yet been clarified due to very limited observed data, compared with atmospheric radiocaesium data. Here, we first revealed that the ratios of ^131^I (decay-corrected to March 11, 2011) to ^137^Cs in radioactive plumes were divided into three groups (A, B, and C) by analysing all published data on atmospheric ^131^I concentrations independently measured immediately after the accident in eastern Japan. Groups A and C were found regardless of whether the measurement sites were located in eastern Fukushima or Kantou areas, while group B was observed only in the eastern Kantou area. The ratios in group A were approximately equal to 10 for the plumes on March 15, March 20, and on the morning of March 21, and those in group B were approximately 75 on March 16. Their possible sources were Unit 2 and/or Unit 3. In contrast, the ratios in group C were approximately equal to 360, much higher than those of groups A and B, and were observed from the afternoon of March 21 to March 25. These high ^131^I concentrations could be released after water supply to FD1NPP.

## Introduction

The atmospheric distributions of radioiodine in the early period after the Fukushima Daiichi Nuclear Power Plant (FD1NPP) accident have not been clarified yet, because data on atmospheric ^131^I concentrations measured in the early period after the accident are very limited. In contrast, there are many data^1–4^ for the spatial distributions of the ^131^I deposition density measured within 3 months after the FD1NPP accident. They clearly showed that a high level zone of ^131^I deposition extended as far as 40 km northwest from the FD1NPP due to precipitation, which was similar to that of ^134^Cs or ^137^Cs deposition. In the case of no precipitation, however, the contribution of atmospheric radioiodine in radioactive plumes was not included in the deposition data except for dry deposition. As a result, estimates of internal radiation doses from inhalation by using ^131^I deposition data still have large uncertainty. Meanwhile, the spatio-temporal distributions of hourly atmospheric ^137^Cs concentrations have been retrieved by using filter-tapes installed in suspended particulate matter (SPM) monitors operated at the air quality monitoring stations in eastern and central Japan^5–8^. By analyzing these measurement data, nine major radioactive plumes (P1-P9) with high ^137^Cs concentrations were identified^5^ (Table S1 and Fig. S1). After that, Nakajima *et al*.^9^ simulated atmospheric ^137^Cs concentrations by using two atmospheric transport models, compared with these measured ^137^Cs concentrations^5,6^, and their transport pathways from the FD1NPP were shown in Fig. S2. Furthermore, hourly atmospheric ^137^Cs concentrations were measured in the SPM collected at two SPM stations (Futaba and Naraha in Fig. S3) located within 20 km from the FD1NPP. By the time-series analysis of hourly ^137^Cs concentrations (Fig. S4), 16 plumes were identified including the nine plumes (Table S1). However, the ratios of ^131^I to ^137^Cs and the dynamics of atmospheric ^131^I in major plumes have not been clarified.

**Figure 1:**
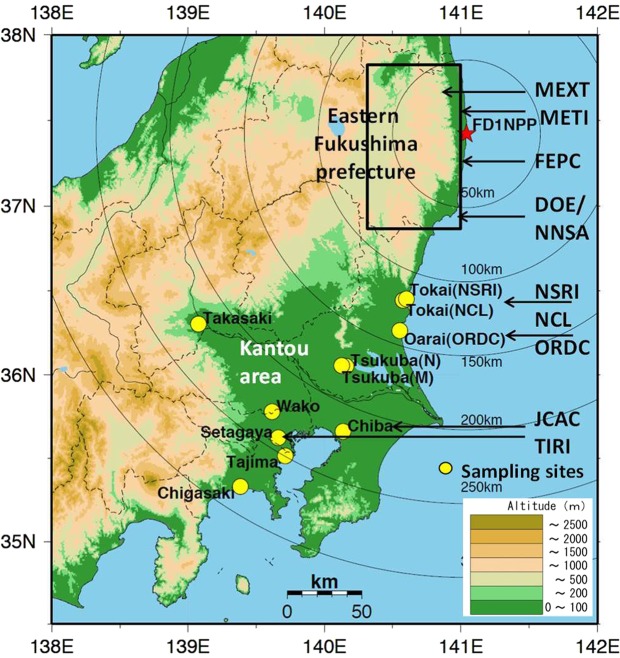
Map of the sampling sites or areas for atmospheric nuclides in the early period after the accident. Detailed information is listed in Table 1. In the Kantou area, atmospheric radionuclides were measured at 11 sites, and atmospheric ^137^Cs and ^131^I concentrations at NSRI, JCAC, and TIRI were analysed in detail in this study. The sampling sites of Tsukuba(N) and Tsukuba(M) are NIES and MRI in Table 1, respectively. The sampling area for the eastern Fukushima prefecture is also shown, and each sampling point by MEXT, FEPC, DOE/NNSA, and METI is shown in Figs 6 and S5, respectively. The mapping was made by using the Generic Mapping Tools (GMT)^50^ and the topography data of USGS EROS Archive - Digital Elevation - Global 30 Arc-Second Elevation (GTOPO30)^51^.

The limited measurements and their sampling properties for atmospheric ^131^I concentrations^10–25^ are shown in Table 1, and their sampling sites in the eastern Fukushima and Kantou areas are shown in Fig. 1, and the radionuclides data are available to the public. According to a preliminary analysis of some datasets in the Kantou area, particulate ^131^I and ^137^Cs concentrations for one day sampling increased on March 15–16, March 20–21, and March 22–23, 2011, and the ratios of ^131^I to ^137^Cs were qualitatively different among these three periods^26^. World Meteorological Organization (WMO) simulated ^137^Cs deposition, and atmospheric ^137^Cs and particulate ^131^I concentrations by several atmospheric transport and deposition models during March 11–31, 2011^27^, and these atmospheric concentrations were compared with those measured by Japan Atomic Energy Agency (JAEA) in Tokai. Lebel *et al*.^28^ analyzed the some datasets to obtain key insights about the different chemical forms. Katata *et al*.^29^ also used several datasets for source term estimation. Hirose^30^ introduced some data on atmospheric ^131^I concentrations measured in Japan and around the world. However, the ratios of ^131^I/^137^Cs in the major plumes, which were identified by the analysis of the spatio-temporal distribution of atmospheric ^137^Cs^5,7,8^ (Table S1 and Figs S1–S4), have not been studied yet, and the dynamics of atmospheric ^131^I has not been clarified. The purpose of this study is to clarify the whole picture for the high atmospheric ^131^I concentrations in major plumes in the early period after the accident by analyzing limited data, to contribute to re-construct radioiodine release rates in major plumes, to improve the atmospheric transport and deposition models for radioiodine, and to re-evaluate internal exposure from inhalation. Furthermore, to understand atmospheric concentrations of not only particulate radioiodine but also gaseous radioiodine in major plumes is essential to the assessment for thyroid cancer.

**Table 1:** Properties of published atmospheric ^131^I measurements in the early period after the Fukushima accident.

Measurement institute/agency	Abbreviation	Sampling site/area	One sampling time (Sampling interval per day)	Sampling materials	References
Headquarters for disaster control of Fukushima pref. Ministry of Economy, Trade and Industry	METI	Area within 2–35 km from FD1NPP	10–20 min. (a few times)	particulates + volatiles	^10^
Ministry of Education, Culture, Sports, Science and Technology	MEXT	Eastern Fukushima Pref.	10–20 min. (1–2)	particulates + volatiles	^11^
US Department of Energy/National Nuclear Safety Agency	DOE/NNSA	Eastern Fukushima Pref.	10–20 min. (1–2)	particulates, volatiles	^12^
Tokyo Electric Power Company	TEPCO	FD1NPP, FD2NPP	8–20 min. (1–2)	particulates, volatiles	^13^
The Federation of Electric Power Companies of Japan	FEPC	Eastern Fukushima Pref.	10–20 min. (2)	(particulates + volatiles)^a^	^14^
JAEA-Nuclear Science Research Institute	NSRI	Tokai, Ibaraki Pref.	20 min. (Several times)	particulates, volatiles	^15^
JAEA-Nuclear Fuel Cycle Engineering Laboratories	NCL	Tokai, Ibaraki Pref.	3–12 hours (2–3)	particulates, volatiles	^16^
JAEA-Oarai Research and Development Center	ORDC	Oarai, Ibaraki pref.	3–12 hours at MS3 (2–4)	particulates, volatiles	^17^
Japan Chemical Analysis Center	JCAC	Chiba, Chiba pref.	1 day (1)	(particulates + volatiles)^b^	^18^
National Institute for Environmental Studies/ High Energy Accelerator Research Organization	NIES/KEK	Tsukuba, Ibaraki pref.	3 hours - 2 days (0.5–2)	particulates, volatiles^c^	^19^
Tokyo Metropolitan Industrial Technology Research Institute	TIRI	Setagaya/Komazawa, Tokyo Metropolitan Government	1–2 hours (12–24)	particulates	^20^
Meteorological Research Institute	MRI	Tsukuba, Ibaraki pref.	6 hours - 1 day (1–2)	particulates	^21^
Comprehensive Nuclear Test-Ban-Treaty Organization	CTBTO	Takasaki, Gunma pref.	1 day (1)	particulates	^22^
RIKEN Wako Campus	RIKEN	Wako, Saitama pref.	1 day (1)	particulates	^23^
Kawasaki Municipal Research Institute for Environment Protection/Osaka University	KMRIEP	Kawasaki/Tajima, Kanagawa pref.	1 day (1)	particulates	^24^
Kanagawa Prefectural Institute of Public Health	KIPH	Chigasaki, Kanagawa pref.	1 day (1)	particulates	^25^

^a^Data of only ^131^I is available.

^b^Three samples were separately measured for gaseous ^131^I(g) and aerosol ^131^I(a).

^c^Two carbon fiber filters without TEDA were set after the first quartz fiber filter.

Among the data listed in Table 1, we analysed the data matched to the following two principles, in order to clarify the whole picture of atmospheric ^131^I in the major plumes. The first principle is that a series of sampling and radionuclide measurement were separately made for particulate/aerosol radioiodine (^131^I(a)) and volatile/gaseous radioiodine (^131^I(g)) collected on the first and second filters, respectively, and that TEDA (triethylenediamine) was impregnated on the second filter to improve the collection efficiency of gaseous ^131^I(g). Another principle is that the sampling interval was made within a few hours, even though it was only for particulate sampling. In this study, we used ^131^I data collected both on the first filter for ^131^I(a) and on the second filter for ^131^I(g). In contrast, we used the ^137^Cs data collected only on the first filter, although Lebel *et al*.^28^ used the data of ^137^Cs on both the first and second filters, and the penetration rates were calculated to re-evaluate ^131^I(g) and ^131^I(a). The difference between our study and their study^28^ will be discussed in detail later. In addition, the serious effects of the radioactive plumes with high radionuclides on environmental contamination in the measurement/sampling system were clearly observed at some sites^15,22^. We also discuss later on the data which were likely contaminated. In our analysis, all ^131^I concentrations were decay-corrected (symbol of [*] is used) to normalize all data measured on different days, because the half-life time of ^131^I is only 8 days. The measurement periods of the data which we analyzed in this study are shown in Table S2.

The data which we mainly used in the Kantou area were those measured by Japan Atomic Energy Agency (JAEA)-Nuclear Science Research Institute (NSRI)^15^ and JAEA-Nuclear Fuel Cycle Engineering Research Laboratories (NCL)^16^ in Tokai, Japan Chemical Analysis Center (JCAC)^18^ in Chiba, and the old Komazawa branch of the Tokyo Metropolitan Industrial Technology Research Institute (TIRI)^20^ in Tokyo. On the other hand, In the eastern Fukushima area, we used the data measured by the Nuclear Emergency Response Headquarters of the Fukushima prefecture and the Ministry of Economy, Trade and Industry (METI)^10^, the Ministry of Education, Culture, Sports, Science and Technology (MEXT)^11^, the U.S. Department of Energy (DOE)/National Nuclear Safety Agency (NNSA)^12^, the Tokyo Electric Power Company (TEPCO)^13^, and the Federation of Electric Power Companies (FEPC)^14^.

## Results

### Ratios of ^131^I(a + g)* to ^137^Cs in the Kantou and eastern Fukushima areas

**Figure 2:**
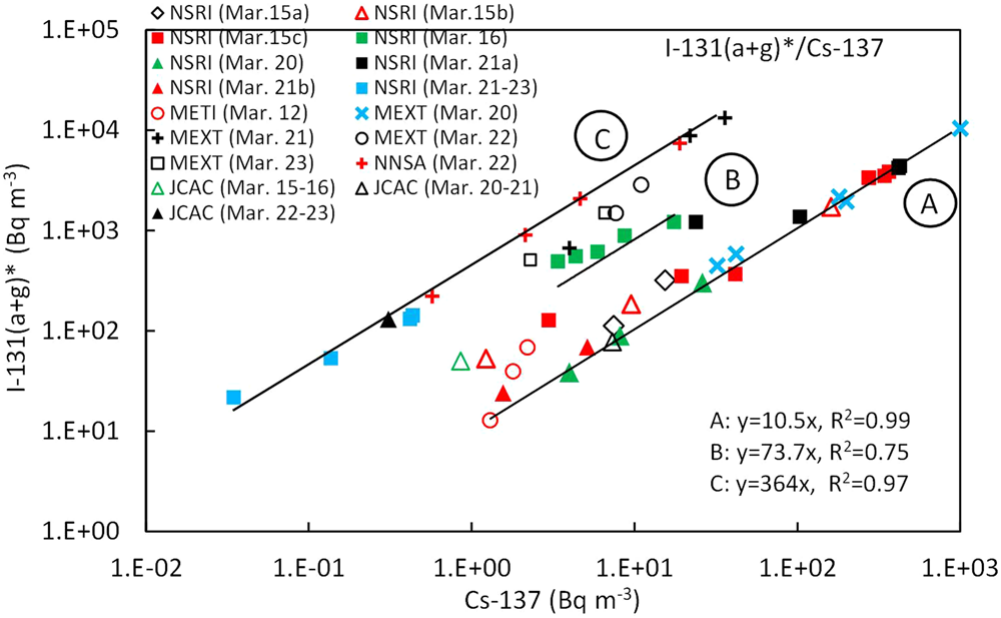
Relationship between ^137^Cs and ^131^I(a + g)* in the Kantou and eastern Fukushima areas. See the text for three groups (**A**–**C**). The measurement periods at NSRI^15^ are shown in Fig. 4a and Table S2. The data of March 15c, March 16, March 20, and March 21a were measured when plumes P2, P4, P7, and P9 arrived, respectively. The measurement points and periods at METI^10^ are shown in Fig. S5 and Table S2, respectively. The measured points and periods on the afternoon of March 20–23 at several sites by MEXT^11^ are shown in Fig. 6 and Table S2, respectively. The measurement points and periods on the morning of March 22 at sites D-G of DOE/NNSA^12^ are shown in Fig. 6 and Table S2, respectively. The measurement periods of March 15–16, March 20–21, and March 22–23 at JCAC^18^ are shown in Fig. 4c and Table S2.

The relationship between the ^131^I(a + g)* and ^137^Cs concentrations in the Kantou and eastern Fukushima areas is shown in Fig. 2. The ratios of ^131^I(a + g)*/^137^Cs are divided into three groups A, B, and C, and the summary is shown in Table 2. According to these results, the ratios did not depend on the Fukushima or Kantou areas, but on the plumes/polluted air masses. Most of the ratios in group A during March 15–21 (except for March 16) were approximately equal to 10, which was nearly equal to the inventory data of 9.2 for Unit 2 and 9.7 for Unit 3 calculated by Nishihara *et al*.^31^. This strongly suggests that plumes P2, P7, P8, and P9 (Figs S1 and S2) were transported towards the sampling sites in Tokai and northwest of Fukushima from the FD1NPP with almost no change in the original ratios from Unit 2 and/or Unit 3 during transport. In contrast, the ratios in group B measured at NSRI^15^ in Tokai on March 16 were approximately equal to 75, which was much higher than in group A. And the ratios were caused by plume P4 (Figs S1 and S2), the center of which passed through the east side of NSRI from the north to the south^5,9^. Two possible reasons as to why group B had much higher ratios compared to those in the inventory data^31^ are considered as follows. The ratio of the radionuclide composition might have already changed in the reactor units before the radionuclides were released into the atmosphere because white smokes were observed from Unit 3 on the morning of March 16^32^. The other reason is that plume P4 might have been affected by wet deposition through precipitation after release into the atmosphere^33^. However, it is currently still unknown which process was dominant. The ratios in group C were approximately equal to 360, which were much higher than those in groups A and B. These values were found at several points by MEXT located south of the FD1NPP on the afternoon of March 21^11^, and were also found at several points by DOE/NNSA located south of the FD1NPP on the morning of March 22^12^. Furthermore, the ratios measured at NSRI^15^ in Tokai during the evening of March 21–23 were almost equal to those found in the eastern Fukushima area, although the ^137^Cs concentrations were less than 1 Bq m^−3^. In the Kantou area, measurements were also carried out at JCAC^18^ in Chiba, which is located 100 km south-southwest of NSRI (Fig. 1), although the sampling interval was one day. The ratios measured on March 15–16, 20–21, and 22–23 were also divided into the same three groups as those at NSRI (Fig. 2). This strongly suggests that these three groups can be found not only at NSRI but also in the broader Tokyo Metropolitan area. Hence, we revealed, for the first time, that the measurement sites for groups A and C were located over a broad area, including not only the eastern Fukushima area but also the Kantou area. Therefore, a more detailed analysis between ^131^I and ^137^Cs concentrations in the Kantou area will be described later, followed by the Fukushima area.

**Table 2:** Summary of the ratios of ^131^I(a + g)*/^137^Cs and ^131^I(a)*/^131^I(a + g)* for the three groups.

Group	Day (plume No.)	Area	Max. ^131^I(a + g)	^131^I(a + g)*/^137^Cs	^131^I(a)*/^131^(a + g)*
			(Bq m^−3^)		
A	March 15 (P2)	Kantou^a^	2,800	10	0.56^b^ (0.48–0.64)
	March 20 (P7)	Kantou	140		
	March 20 (P8)	Fukushima	4,800		
	AM of March 21(P9)	Kantou	1,920		
B	March 16 (P4)	Kantou^a^	812	75	0.28^c^ (0.24–0.34)
C	PM of March 21-	Fukushima	5,600	360	0.41^d^ (0.18–0.55)
	PM of March 21-	Kantou	53.5		

^a^Published data only in the Kantou area were available.

^b^Average of the ^131^I(a + g) concentrations higher than 1,000 Bq m^−3^ measured by NSRI.

^c^Average of the data by NSRI.

^d^Average of the data by NSRI and JCAC.

### Ratios of particulate ^131^I(a)* to the total ^131^I(a + g)* in the Kantou area

**Figure 3:**
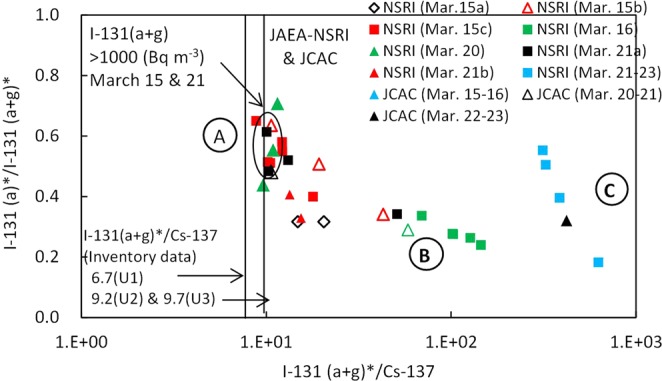
Relationship between ^131^I(a + g)*/^137^Cs and ^131^I(a)*/^131^I(a + g)* in the Kantou area. The measurement periods at NSRI^15^ and JCAC^18^ are the same as those in Fig. 2. The data of the measured ^131^I(a + g) concentrations more than 1,000 Bq m^−3^ at NSRI on March 15 and March 21, 2011, are shown in the circle. The ^131^I(a + g)*/^137^Cs values in the inventory data^31^ are also shown.

Analysing the data in which particulate/aerosol ^131^I(a)* and gaseous/volatile ^131^I(g)* were measured at NSRI in Tokai and JCAC in Chiba, each of the three groups (A, B, and C) had different ratios (=Y) of particulate/aerosol ^131^I(a)* to total ^131^I(a + g)* as shown in Fig. 3 and Table 2. The average ratio of Y in the A group was 0.56 (0.48–0.64), among the data at NSRI^15^ in which the ^131^I(a + g) concentrations were higher than 1,000 Bq m^−3^ in plumes P2 and P9. As a result, the aerosol ^131^I(a)* concentrations were equal to or slightly higher than the gaseous ^131^I(g)* concentrations. In contrast, the average ratio of Y in the B group was 0.28, indicating that the gaseous ^131^I(g)* concentrations were much higher than the aerosol ^131^I(a)* concentrations. In addition, the average ratio of Y in the C group was 0.41, and higher than that in group B but less than that in group A. Furthermore, the ratios of Y for three data at JCAC^18^ were similar to those in the three groups. Hence, this relationship was estimated to be obtained in all the Kantou area, because the two sites of NSRI and JCAC are located in the eastern and central parts of the Kantou area, respectively. However, it is still unknown whether or not the ratios of Y can be also applied to those for radionuclides just after released into the atmosphere from the FD1NPP, because the measurement sites of NSRI and JCAC are located far from the FD1NPP.

### Relationship between ^131^I and ^137^Cs in the Kantou area

**Figure 4:**
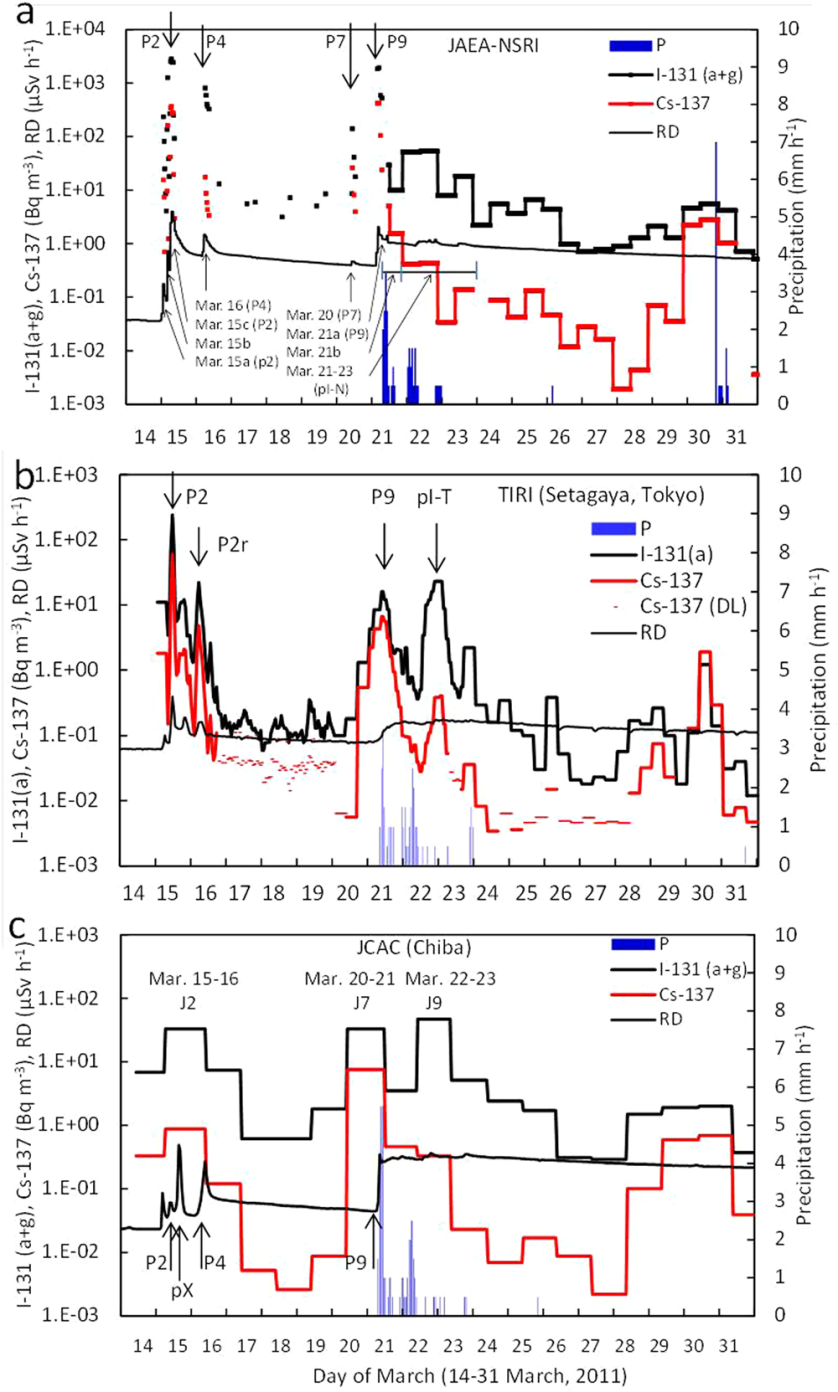
Time series of atmospheric concentrations of ^137^Cs and ^131^I(a + g) or ^131^I(a), precipitation (P), and radiation dose rate (RD) during March 14-31, 2011, at (**a**) NSRI, (**b**) TIRI, and (**c**) JCAC. The measurement periods at NSRI^15^ and JCAC^18^ are the same as those in Fig. 2. Plume numbers of P2, P4, P7, and P9 are listed in Table S1^8^. Plumes with extremely high ratios of ^131^I/^137^Cs in group C are shown as pI-T at TIRI^20^ during March 22–23 and pI-N at NSRI during March 21–23, which are described in the text. Because DL for ^137^Cs at TIRI means a value of detection limit, the ^137^Cs concentration at the time was not detected.

**Figure 5:**
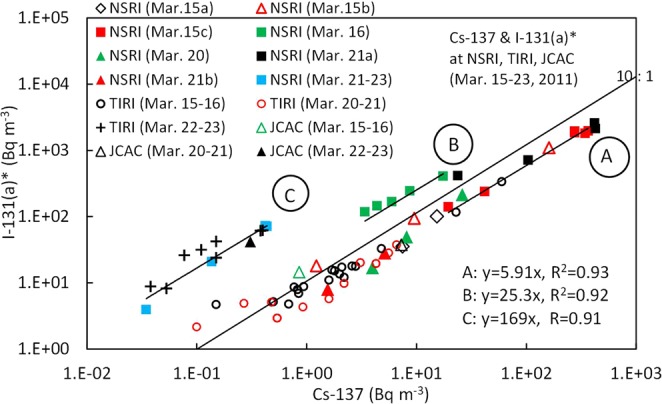
Relationship between ^137^Cs and aerosol ^131^I(a)* in the Kantou area. The measurement periods at NSRI^15^, TIRI^20^, and JCAC^18^ are shown in Fig. 4 and Table S2. The periods of Mar. 15–16, Mar. 20–21, and Mar. 22–23 at TIRI corresponded to those of high ^131^I(a) concentrations including plumes P2, P9, and pI-T in Fig. 4b, respectively.

Time series of atmospheric ^131^I(a + g) and ^137^Cs concentrations at NSRI^15^ in Tokai, TIRI^20^ in Tokyo (only ^131^I(a) concentrations), and JCAC^18^ in Chiba during March 14–31, 2011, are shown in Fig. 4. The peaks of plume P2 on March 15 and plume P9 on March 21 were observed at both of NSRI and TIRI, while the highest ^137^Cs concentration at the peak time on the morning of March 21 at TIRI was much lower than that at NSRI, suggesting that TIRI would be located near the western edge of plume P9. In contrast, the peak of plume P4 was observed at NSRI and JCAC on March 16, and the ^131^I(a + g) concentrations were much higher than the ^137^Cs concentrations compared with those on March 21. At TIRI, however, plume P4 was not found on the morning of March 16, because a northerly wind prevailed in the western Kanto area. A peak of P2r in Fig. 4b observed at TIRI during 5:00–7:00 (JST) on March 16 was caused by part of the aged polluted air masses with high radionuclides returned back to the south, which had been transported to the western or northern Kanto areas as plume P2 on the afternoon of Mach 15^26^. Furthermore, the ^131^I(a + g) concentrations at NSRI during March 21–23 (pI-N in Fig. 4a) were much higher than the low ^137^Cs concentrations (<1 Bq m^−3^). At TIRI, plume pI-T with high particulate ^131^I(a) concentrations with the maximum of 23 Bq m^−3^ was observed during March 22–23, 2011 (Fig. 4b), although the maximum ^137^Cs concentration was 0.4 Bq m^−3^, resulting in a high ratio of ^131^I(a)*/^137^Cs of 155. Although the ^131^I and ^137^Cs concentrations at JCAC were not as high as those at NSRI and TIRI due to the one-day sampling interval (Fig. 4c), the relationship between ^131^I(a + g) and ^137^Cs was similar to that at NSRI. These high ratios in the C group have not been focused until now, possibly because the ^137^Cs concentrations were lower than 1 Bq m^−3^ in the Kantou area. Then, the relationship between ^137^Cs and aerosol ^131^I(a)* at TIRI was compared with that at NSRI for plumes P2, P9, and pI-T. The ratios of ^131^I(a)*/^137^Cs in plumes P2 and P9 at TIRI, which are the data of Mar. 15–16 and Mar. 20–21 in Fig. 5, respectively, were almost equal to those at NSRI in the A group, and the ratios in plume pI-T at TIRI (Mar. 22–23 in Fig. 5) were also equal to those at NSRI in the C group. The ratios of the three data points at JCAC also shown in Fig. 5 were almost equal to those at NSRI and TIRI, which is located approximately 40 km west of JCAC. Considering that the equilibrium state for the relationship between ^137^Cs and the aerosol ^131^I(a)* was obtained at these three sites, it is reasonable that the ratios of ^131^I(a)*/^131^I(a + g)* were also estimated to be the same values at these three sites as well as the ratios of ^131^I(a + g)*/^137^Cs.

### Relationship between ^131^I(a + g)* and ^137^Cs in the eastern Fukushima prefecture

**Figure 6:**
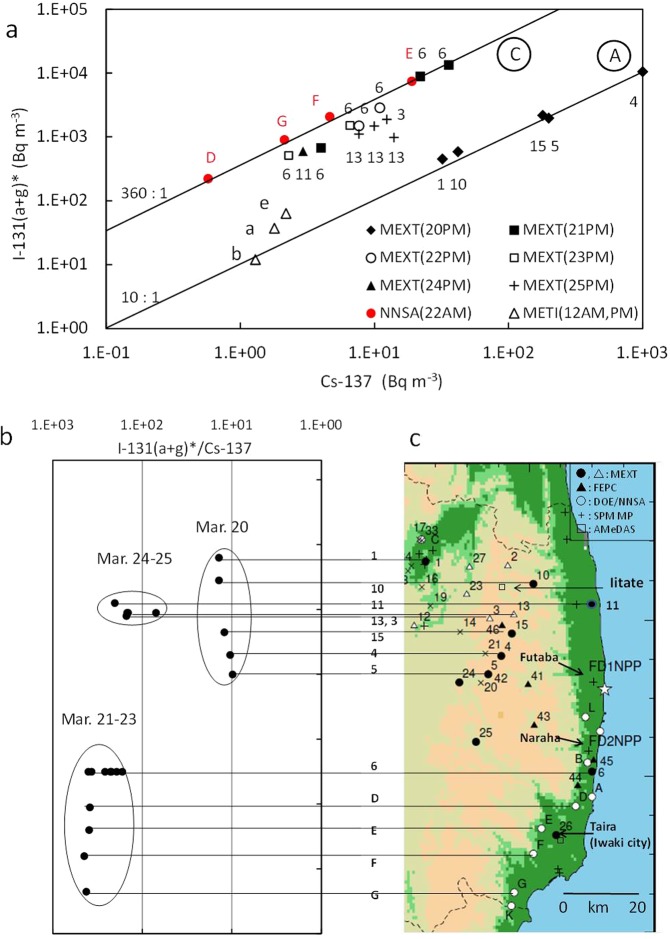
Spatial distributions of ^137^Cs, ^131^I(a + g)* and ^131^I(a + g)*/^137^Cs in the eastern Fukushima area. (**a)** Relationship between ^137^Cs and ^131^I(a + g)* measured by METI^10^ on March 12, by MEXT^11^ on March 20–25, and by DOE/NNSA^12^ on the morning of March 22. The measurement periods are shown in Table S2. The measurement points are shown with numerical or alphabetical numbers, which are found in (**c**). **(b)** The ^131^I (a + g)*/^137^Cs values at measurement points in two groups A and C are shown, which were measured on March 20, March 21–23, and March 24–25. **(c)** The measurement points of 1–15, 41–46, and D-G at MEXT, FEPC, and DOE/NNSA, respectively, are shown on a map of the eastern Fukushima prefecture. The SPM monitoring stations (SPM MP) of Futaba and Naraha operated by the Fukushima prefecture are also shown. Furthermore, the AMeDAS meteorological stations of Iitate and Taira by JMA^34^ are shown. The AMeDAS station of Taira by JMA^34^ in Iwaki city and the environmental radioactivity monitoring point of Taira by Fukushima prefecture are located near the monitoring point of 26 by MEXT. The mapping of c was made by using the Generic Mapping Tools (GMT)^50^ and the topography data of USGS EROS Archive - Digital Elevation - Global 30 Arc-Second Elevation (GTOPO30)^51^.

The spatial distributions in the Fukushima area during March 20–25, 2011, were analyzed in detail based on the data measured by MEXT^11^ and DOE/NNSA^12^ in groups A and C in Fig. 2, using the observed ^131^I (a + g) concentrations higher than approximately 100 Bq m^−3^. By picking up the data by MEXT and DOE/NNSA from Fig. 2 and adding the data measured by MEXT on March 24–25, a scatter diagram was created between the ^137^Cs and ^131^I(a + g)* concentrations in the eastern Fukushima area. As shown in Fig. 6, five data by MEXT in group A with the high ^131^I(a + g) concentrations (203–4,800 Bq m^−3^) were measured at 5 sites (Nos. 4, 15, 5, 10, and 1 in Fig. 6) located 30–60 km west/northwest of the FD1NPP on the afternoon of March 20 when plume P8 arrived by a southeasterly wind^5,7,9^ (Table S1, Figs S1 and S2). In addition, the high ^131^I(a + g) concentrations (970–4,100 Bq m^−3^) were measured by FEPC^14^ at three sites (Nos. 41, 42, and 46 in Fig. 6) located near the three sites (Nos. 4, 15, 5) by MEXT on the morning or afternoon of March 20, although the ^137^Cs data were not available. This strongly supports the existence of plume P8 in a wide area, indicating that the center of P8 was estimated to be located near site 4. However, at the sites closely located south of these high ^131^I(a + g) sites, the ^131^I(a + g) concentrations were very low (33.7 Bq m^−3^) at site 24 by MEXT, and below the detection limit at site 43 by FEPC^14^, indicating that the west boundary of plume P8 was estimated to be located between the sites with the high and low ^131^I(a + g) concentrations.

In contrast, group C had two datasets with high ^131^I(a + g) concentrations independently measured by MEXT^11^ and DOE/NNSA^12^ during March 21–25, 2011 (Fig. 6). On the afternoon of March 21, at site 6 by MEXT, which was located 23 km south of the FD1NPP, the highest ^131^I(a + g) concentration of 5,600 Bq m^−3^ was observed and the ratio of ^131^I(a + g)*/^137^Cs was approximately 370, extremely higher than the ratios measured in the northwest area one day prior (Fig. 6). Following the highest ^131^I(a + g) concentration and ratio by MEXT, at 4 sites (D, E, F, and G in Fig. 6) by DOE/NNSA^12^, which were located 35–65 km south of the FD1NPP, the highest ^131^I(a + g) concentration of 2,961 Bq m^−3^ was observed on the morning of March 22, and the ratios were as high as the ratios measured at site 6 one day prior (Fig. 6). At site 6, the high ^131^I(a + g) concentrations of 110–1,100 Bq m^−3^ were still observed on the afternoons of March 22 and 23 although the ratios gradually decreased to 193–260 (Fig. 6). Furthermore, on the afternoons of March 24 and 25, the high ^131^I(a + g) concentrations of 193–555 Bq m^−3^ were observed at site 11 by MEXT, which was located 25 km north of the FD1NPP, and at two sites 3 and 13 by MEXT, which were approximately 35 km of northwest of the FD1NPP, respectively. The ratios of ^131^I(a + g)*/^137^Cs at these three sites were 69–202, much higher than those measured in the same area on March 20. According to the wind directions measured at the Iitate and Iwaki meteorological stations (Figs 6c and S3a) by the Japan Meteorological Agency (JMA)^34^ and at the FD2NPP by TEPCO^35^, southeasterly winds and southerly winds observed on March 20 (Fig. S1c) and March 24, respectively, and northwesterly or northeasterly winds prevailed during March 21–23 in the eastern Fukushima area. As a result, the polluted air masses with high ^131^I(a + g) concentrations measured in the eastern Fukushima prefecture during March 20–25 were guessed to be directly transported from the FD1NPP by those wind systems, though detailed model simulations should be studied to confirm this point.

These results strongly suggest that the release rates of ^131^I and ^137^Cs from the FD1NPP drastically changed between the morning and afternoon of March 21, 2011, because the ratios were nearly equal to 10 in plume P9 measured at NSRI in the Kantou area on the morning of March 21, as previously described. A possible reason why the ratio became so high on the afternoon of March 21 will be discussed later. According to the data by FEPC^14^, a high ^131^I(a + g) concentration of 1,400 Bq m^−3^ was observed at 11:00 (JST) on March 21 at site 44 (Fig. 6), which was located approximately 7 km southwest of site 6 by MEXT. However, a ^131^I(a + g) concentration of 80 Bq m^−3^ was observed at approximately 11:00 on March 21 at site 45, located 10 km northeast of site 44, which was much lower than that at site 44. Meanwhile, at the Naraha SPM station (Figs 6 and S3), which is located 10 km north-northeast of site 44, a high ^137^Cs concentration of 106 Bq m^−3^ was observed at 9:00 (JST) on March 21, due to the transport of plume P9′ from the FD1NPP^8^ (Table S1 and Fig. S4), while the plume was not transported to the Kantou area^8^. Therefore, the polluted air masses measured at site 44 at 11:00 on March 21 were possibly part of plume P9′ because site 44 was located on the downwind side of the Naraha station. Plume P9′ would be emitted from the FD1NPP under a transition period of the drastic change in the emission rates of ^131^I(a + g) in the FD1NPP, although the ^137^Cs concentrations at site 44 by FEPC^14^ were not available.

During March 12–13, 2011, one or two days after the accident, the Fukushima prefecture and METI measured atmospheric radionuclides near the FD1NPP^10^ (Fig. S5). On March 12 when the radionuclides first released from Unit 1, both ^131^I and ^137^Cs concentrations were detected only for three data points (a, b, and e in Fig. 6a), although the other ^137^Cs data were below the detection limits (Fig. S5). At these three data points, ^131^I(a + g) concentrations were in the range of 12–63 Bq m^−3^ and the ^137^Cs concentrations were 1–2 Bq m^−3^. Accordingly, the ratios of ^131^I(a + g)*/^137^Cs were in the range of 10–30, which are much higher than the ratio of 6.7 for Unit 1 in the inventory data^31^. Possible reasons why the ratios were higher than the ratio for Unit 1 in the inventory data^31^ could be due to water injection from 4:00 (JST) and/or the opening operations of the small S/C vent valve from 10:17 (JST) on March 12^36^, although the uncertainties are still large because it has not been clarified whether these works were partly successful or not. It is noted that the maximum ^131^I(a + g) concentration was 165 Bq m^−3^ with the ^137^Cs concentration below the detection limit (Fig. S5). At the Futaba SPM station (Figs S3 and S4), 3.2 km northwest of the FD1NPP, the ^137^Cs concentrations with a maximum of 55.7 Bq m^−3^ at 9:00 (JST) were observed on the morning of March 12 due to an early release of radionuclides from Unit 1^8^. These polluted air masses would be directly released from the FD1NPP.

### A new emissions event with extremely high ratios of ^131^I/^137^Cs during March 21–25, 2011

These analyses in group C strongly suggest a new emissions event from the FD1NPP with extremely high ratios of ^131^I(a + g)*/^137^Cs. The first observational data were found at site 6 by MEXT (Fig. 6) when a northeasterly wind was blowing during March 21–23, 2011, and the highest concentration of ^131^I(a + g) was 5,600 Bq m^−3^, which was very high compared to the ^137^Cs concentration of 36 Bq m^−3^ on the afternoon of March 21^11^. The second observational data, in which the highest ^131^I(a + g) concentration was 2,961 Bq m^−3^ with the ^137^Cs concentration of 19.0 Bq m^−3^, were found on the morning of March 22 at site E by DOE/NNSA^12^, which was located 20 km southwest of site 6 due to a northeasterly wind. Following these data, high ratios were still found at site 11 in the northern area and sites 3 and 13 in the northwestern area from the FD1NPP on March 24 and 25, respectively. The ratios at these sites became gradually lower than those in the first and second highest ^131^I(a + g) data (Fig. 6). These results indicate that the release rate of radioactive materials from the FD1NPP gradually decreased day by day. On the other hand, in the Kantou area, the ratios of ^131^I(a + g)*/^137^Cs with an average of approximately 340 (except for the lower ^137^Cs concentration less than 0.1 Bq m^−3^) in group C were observed at NSRI from 21:10 on March 21 to 21:00 on March 23, while the ratio before 21:10 on March 21 was only 15^15^ (Fig. 2 and pI-N in Fig. 4a). The other data with high ratios of ^131^I(a)*/^137^Cs (more than 160) were found at TIRI in central Tokyo from the afternoon of March 22 to the morning of March 23 (pI-T in Fig. 4b and group C in Fig. 5), although only the aerosol ^131^I(a) concentrations were measured^20^, as were the ratio observed in Chiba by JCAC^18^. Furthermore, the atmospheric ^137^Cs concentrations at many SPM stations in the Tokyo metropolitan area showed a small peak (approximately 1 Bq m^−3^) on the night of March 22^6^ (Fig. S6), which was similar to the peak of pI-T at TIRI. These data would be affected by the transport of polluted air masses released into the atmosphere from the FD1NPP after March 21. Considering all these data, a new emissions event with the extremely high ratios of ^131^I(a + g)*/^137^Cs possibly started on the late morning of March 21, 2011, and continued at least until March 25 when the ratios were still high in the northwestern area of the FD1NPP (Fig. 6). A possible mechanism for a large amount of radioiodine production in the FD1NPP would be caused by the direct effect of water supply to the FD1NPP for a few or several days, as Hidaka and Ishikawa^37^ estimated radioiodine release due to accumulated water. However, it is unknown which units were responsible for this event. This mechanism should be studied in detail in the future.

### Atmospheric ^137^Cs and ^131^I(a + g) concentrations at the FD1NPP and FD2NPP sites

**Figure 7:**
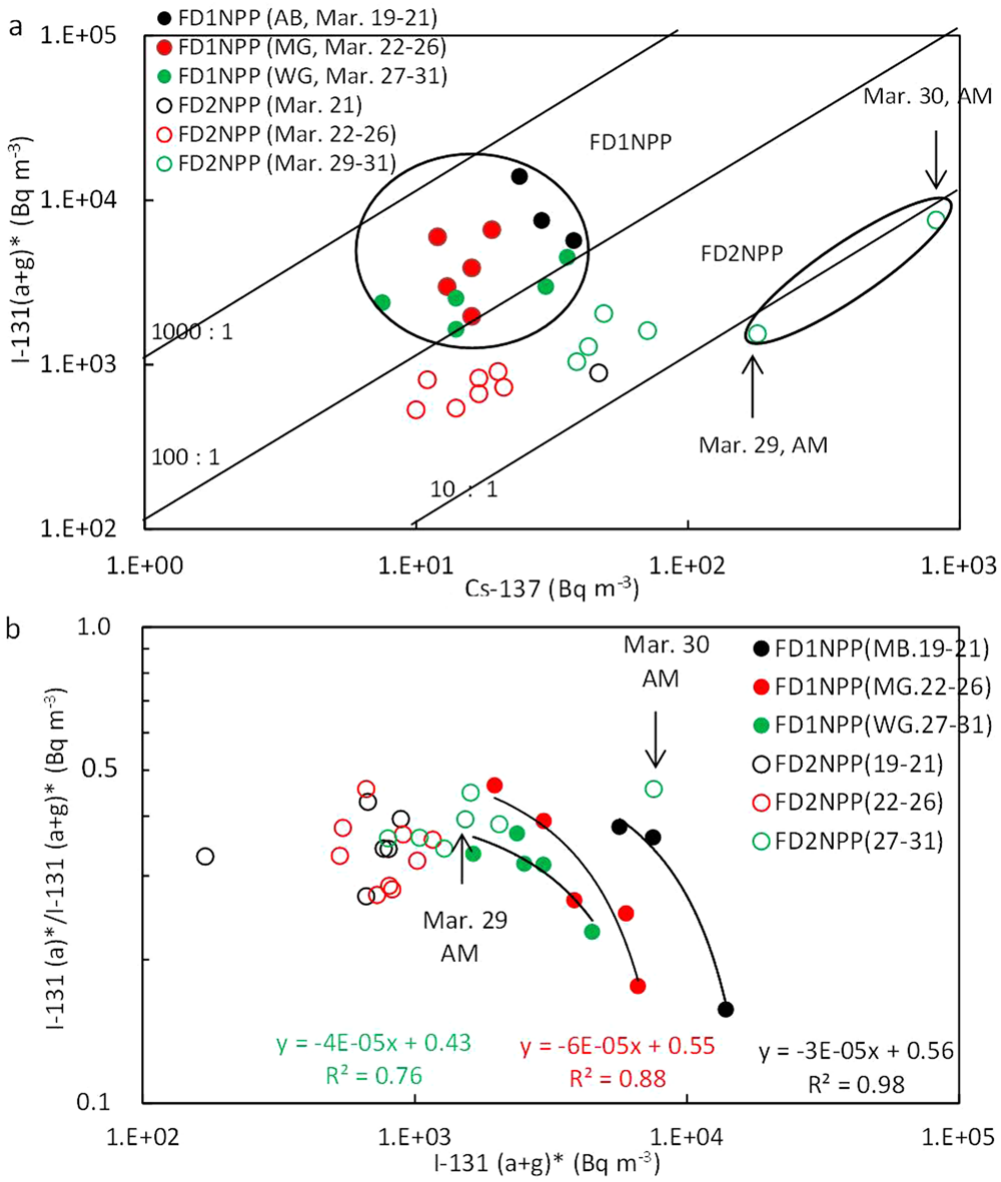
Relationships among ^137^Cs, ^131^I(a + g)*, and ^131^I(a)*/^131^(a + g)* at the sites of FD1NPP and FD2NPP. The measurement periods at FD1NPP and FD2NPP by TEPCO^13^ are shown in Table S2. **(a)** Relationship between ^137^Cs and ^131^I(a + g)*. The ^137^Cs concentrations at FD2NPP during March 19–20 and March 27–28 were below the detection limits. **(b)** Relationship between ^131^I(a + g)* and ^131^I(a)*/^131^I(a + g)*. The monitoring points at the site of FD1NPP were the north point of the administration building (AB), main gate (MG), and west gate (WG), which were located 0.5 km north, 1.0 km west-southwest, and 1.1 km west of the center between reactor Unit 2 and Unit 3 of FD1NPP, respectively^52^. The monitoring post at the site of FD2NPP was MP-1 which was located 0.5 km north of reactor Unit 4 of FD2NPP.

The ranges of atmospheric ^131^I(a + g) and ^137^Cs concentrations at the FD1NPP during March 19–31, 2011, were 360–7,000 and 7.5–38 Bq m^−3^, respectively^13^ (Fig. 7a). These are much different from the data at NSRI more than 100 km south of the FD1NPP, where the maximum ^131^(a + g) and ^137^Cs concentrations in plume P9 on March 21 were 1,920 and 426 Bq m^−3^, respectively^15^. This indicates that no plumes arrived at the sites of FD1NPP in the periods because the maximum ^137^Cs concentration of 38 Bq m^−3^ was much lower than that at NSRI. In addition, the ratios of ^131^I(a + g)*/^137^Cs were in the range of 100–580 at the FD1NPP sites, much higher than those at the FD2NPP site. Furthermore, the ratios of ^131^I(a)*/^131^I(a + g)* showed that the higher the ^131^I(a + g)* concentrations, the lower the ratios (down to 0.16) (Fig. 7b). This strongly suggests that gaseous ^131^I(g) was dominant when the radionuclides were released possibly by the leaks from the FD1NPP after water was injected into the FD1NPP. However, these results are expected to be carefully re-evaluated after a detailed consideration of uncertainty on the measured ^131^I and ^137^Cs concentrations.

In contrast, at the monitoring site (MP-1) of the FD2NPP, the ^137^Cs concentrations were higher than 100 Bq m^−3^ on the mornings of March 29 and March 30 and the ratios of ^131^I(a + g)*/^137^Cs were 8.5 and 9.2, respectively^13^, which were nearly equal to the inventory data of Unit 2 and/or Unit 3^31^ (Fig. 7a). These figures are the same as the ratios in the A group (Fig. 2), and the plumes would pass through near the FD2NPP. In addition, the ratios of ^131^I(a)*/^131^I(a + g)* were 0.39 and 0.45, which were lower than those in the plumes of the A group at NSRI. If we assume that the gaseous ^131^I(g)* would be dominant when the plumes were released from the FD1NPP, gas to particle conversion would have occurred to some extent during transport from FD1NPP to FD2NPP. These polluted air masses might be transported to the Kantou area on the morning of March 30 when the ^137^Cs and ^131^I(a + g) or ^131^I(a) concentrations were high at NSRI in Tokai and TIRI in Tokyo (Fig. 4), because northeasterly winds were observed at the FD2NPP and NSRI in Tokai.

## Discussion

According to these results, the ratios of ^131^I(a + g)*/^137^Cs in the plumes arrived in the Kantou area are expected to be almost equal to the ratios at NSRI if the environmental conditions did not change significantly. Then, the highest ^131^I(a + g)* concentrations at the SPM stations in the Kantou area can be estimated in many plumes during March 15–23, 2011, using the peak ^137^Cs concentrations in each plume observed at the SPM stations^5,6^, because hourly atmospheric ^137^Cs concentrations in SPM collected on the filter-tapes installed in the SPM monitors, were already measured during March 12–23, 2011.

### Estimate of atmospheric ^131^I(a + g) concentrations in plumes near the FD1NPP on March 12 and 15, 2011

In contrast, the estimate of atmospheric ^131^I(a + g)* concentrations in the Fukushima prefecture before March 19, 2011, has large uncertainty due to the lack of atmospheric ^131^I data. Meanwhile, at the Futaba SPM station, which is located 3.2 km northwest of the FD1NPP (Figs 6 and S3), the highest ^137^Cs concentration of 13,600 Bq m^−3^ was observed in plume P1v at 15:00 JST on March 12, 2011, by the vent operation of Unit 1 before the hydrogen explosion at 15:36 JST^8^ (Fig. S4). As a result, the ratio of ^131^I(a + g)*/^137^Cs was estimated to be much higher than the value in the inventory data (6.7)^31^ because part of ^137^Cs could be removed and part of ^131^I could be evaporated through a suppression chamber. Supposing the ratios of ^131^I(a + g)*/^137^Cs at Futaba were equal to those measured near the FD1NPP on the morning of March 12 by METI^10^, the ^131^I(a + g)* concentration at 15:00 JST was estimated to be in the range of 136,000–408,000 Bq m^−3^. Furthermore, at the Naraha SPM station, which is located 17.5 km south of the FD1NPP (Figs 6 and S3), the highest ^137^Cs concentration of 8,300 Bq m^−3^ was observed in plume P2 at 3:00 JST on March 15, 2011^8^, after the direct release from Unit 2 and/or Unit 3 (Fig. S4). Using the ratio of ^131^I(a + g)*/^137^Cs of 10 in plume P2, the ^131^I(a + g)* concentration at 3:00 JST was estimated to be 83,000 Bq m^−3^. As previously described, this plume was transported southward towards NSRI on the morning of March 15, and the ^131^I(a + g) concentration of 1,920 Bq m^−3^ was observed during 6:00–8:00 JST at NSRI (the data of Mar. 15c in Figs 2 and 4a). If these estimated ^131^I(a + g)* concentrations at Futaba and Naraha are reasonable, the ^131^I(a + g)* concentrations in plumes P1v and P2 which were released into the atmosphere from FD1NPP on March 12 and 15, 2011, respectively, would be much higher than 100,000 Bq m^−3^ in areas near the FD1NPP. Furthermore, the second highest ^137^Cs concentration of 5,530 Bq m^−3^ at Futaba was observed at 14:00 JST on March 20 (plume P8 in Table S1 and Fig. S4)^8^. The ^131^I(a + g)* concentration at the peak time was estimated to be 55, 300 Bq m^−3^, because, as previously described, the ratio of ^131^I(a + g)*/^137^Cs was approximately equal to 10, which was measured by MEXT^11^ in the area located northwest of FD1NPP (Figs 2 and 6). The ratios of particulate ^131^I(a) to the total ^131^(a + g) at the released time, however, are still unknown in this stage.

Hirayama *et al*.^38,39^ calculated the atmospheric ^131^I(a + g) concentrations by using the pulse height distribution of NaI(Tl) detectors at the monitoring posts operated by the Environmental Radioactivity Monitoring Center of the Fukushima prefecture^40^. Among the monitoring posts, the highest radiation dose rate of 1.6 mSv h^−1^ was observed at 15:00 JST on March 12, 2011, at the Kamihatori monitoring post^8,40^, which is located 2.5 km northwest of the Futaba SPM station (Figs S3 and S4). As a southeast wind blew in this area, plume P1v was transported first towards Futaba and then towards Kamihatori^8^. However, the ^131^I(a + g) concentration at this peak time was not available due to the extremely high count rate^38,39^. On the other hand, they calculated the ^131^I(a + g) concentrations at the Matsudate and Futatsunuma monitoring sites, which are located north and south of the Naraha station, respectively (Fig. S3). The ^131^I(a + g) concentrations of 7,200 Bq m^−3^ and 9,500 Bq m^−3^ were calculated at 1:00 JST at Matsudate and at 3:00 JST at Futatsunuma, respectively, on March 15, 2011. These concentrations were much lower than the estimated highest ^131^I(a + g) concentration at the Naraha station. The ^131^I(a + g) concentrations, however, are usually much different among the sites depending on whether the site is located near the center or edge of the plume. On the early morning of March 15, the center of plume P2 possibly passed through near the Naraha station, because the highest radiation dose rate of 146 μSv h^−1^, which was much higher than that at Matsudate and Futatsunuma, was observed at the Yamadaoka monitoring site, which is located very close to the Naraha station^8,40^ (Fig. S3). A detailed discussion on the pathway of the plume is expected by using atmospheric transport models in the future. As Hirayama *et al*.^38,39^ did not calculate the radiocaesium concentrations at the monitoring posts, we did not compare them with the observed ^137^Cs data at the Futaba and Naraha stations. At this stage, the ^131^I(a + g) concentrations near the FD1NPP are still unknown, especially on March 12, 2011. Therefore, another study such as the measurement of ^129^I concentrations using filter-tapes at the SPM sites is needed to estimate the ^131^I(a + g) concentrations near the FD1NPP.

### Effects of atmospheric ^131^I(a + g) concentrations on deposition densities of ^131^I in the eastern Fukushima area

As previously described, the ^131^I deposition densities in soils were much higher in the north and northwest areas from the FD1NPP than those in the south area. However, the deposition ratios of ^131^I to ^137^Cs (or ^134^Cs) were much higher in the south area from the FD1NPP than those in the northwest area^1–4^, as shown in Fig. S7^41^. This is supposed to be caused by the new emissions event. The ratios of ^131^I(a + g)*/^137^Cs in group C were very high at site 6 by MEXT from the afternoon of March 21 to March 23^11^, and at 4 sites (D, E, F, G) by DOE/NNSA on the morning of March 22^12^. According to the amount of precipitation analysed by radar-AMeDAS by JMA^42^, precipitation started from 8:00 (JST) to 17:00 on March 21 in the south area of the FD1NPP (Fig. S8). In addition, the Taira meteorological station in the Iwaki city (Figs 6c and S3a), which is located near the sites of E and F, precipitation was frequently observed from 8:00 on March 21 to 8:00 on March 22^34^ (Fig. S9). Furthermore, the radiation dose rates at the Taira monitoring point by Fukushima prefecture located near the Taira meteorological station showed a maximum soon after precipitation due to plume p9′ (Fig. S9)^43^, indicating that the polluted air masses with high radionuclides concentrations passed around the monitoring point by a northerly wind. The high ^131^I(a + g) concentrations of 1,400 and 1,800 Bq m^−3^ were also measured during 10:50–11:08 and 14:40–14:53 JST on March 21, respectively, at site 44 by FEPC^14^ where precipitation was recorded. Hence, radionuclides emitted from the FD1NPP on March 21 by the new emissions event were transported to the south by northerly winds, and part of them possibly deposited in the south area of the FD1NPP during March 21–22 through these precipitation events, resulting in the high ratios of ^131^I/^137^Cs measured in the surface soils of the south area. Another wet deposition might occur in the south area through the precipitation amount of 2.5 mm at Taira observed during 2:00–4:00 JST on March 16^34^ (Fig. S9) when the radiation dose rates also increased possibly due to the passage of plume P4^43^. This effect on deposition density in the south area, however, is estimated to be minor because the precipitation period was very short, and the radiation dose rates after precipitation were quickly recovered to the low level before the precipitation period. In contrast, the radiation dose rates after a maximum at 11:00 JST on March 21 were much higher than the level before the precipitation period due to ground-shine, which was possibly caused by a large amount of radionuclides deposition (Fig. S9).

## Methods

### Details of published atmospheric ^131^I and ^137^Cs measurements in the early period after the accident

Among all data listed in Table 1, we analysed the ^131^I and ^137^Cs data measured by METI, MEXT, DOE/NNSA, TEPCO, and FEPC in the eastern Fukushima area, and by NSRI, NCL, ORDC, JCAC, and TIRI in the Kantou area located more than 100 km south of the FD1NPP. The data of measurement periods are listed on Table S2, after the careful screening of all data. Due to the short life time of 8 days for ^131^I, we used the decay-collected ^131^I concentrations to the stop time of FD1NPP at 14:46 (JST) on March 11, 2011, and the symbol of [*] was attached. Gaseous and aerosol radioiodine, which were separately collected on the filters except for TIRI, were named as ^131^I(g) and ^131^I(a), respectively.

The key data in our analysis are those measured by NSRI^15^ in Tokai (Fig. 1). Atmospheric radionuclides were collected every 20 minutes when the radiation dose rate increased due to the arrival of plumes during March 15–21, 2011, because the plumes transported to the Kantou area from the FD1NPP were first detected on the early morning of March 15. Accordingly, the ratios of ^131^I(a + g)*/^137^Cs and ^131^I(a)*/^131^I(a + g)* were precisely determined at the peak time in major plumes. From the night of March 21, a sampling duration changed to be 12 hours. In Tokai, the measurements of atmospheric radionuclides were also made by NCL^16^, which is located approximately 2 km south of NSRI. We used these data compared to those measured at NSRI, because the sampling duration by NCL was much longer than that by NSRI until the morning of March 21, 2011. Some data measured at ORDC^17^ were used because the systematic measurement started on March 20, 2011. In the central Kantou area, the gaseous ^131^I(g) and aerosol ^131^I(a) data measured by JCAC^18^ in the Chiba prefecture were also analyzed. Furthermore, the data measured at the old Komazawa branch of the TIRI^20^ were also used, because the aerosol sampling interval by a high volume air sampler was every one or two hours during March 15–23, 2011. In contrast, in the eastern Fukushima prefecture, the Nuclear Emergency Response Headquarters of the Fukushima prefecture and METI^10^ urgently collected gaseous and aerosol radionuclides with sampling times less than 20 minutes at approximately 20 points located within 2–35 km of the FD1NPP on March 12–13, 2011 (Fig. S5). On March 18, 2011, MEXT^11^ started to collect gaseous and particulate radionuclides at many points located within 60 km of the FD1NPP. Discrete sampling was performed several times in the daytime at some points. We also analysed the data with high radionuclide concentrations by DOE/NNSA^12^, who started to measure gaseous and particulate radionuclides on March 17 at many points in the eastern region of Japan including the eastern Fukushima area. TEPCO^13^ also started to measure gaseous and particulate radionuclides on March 19, 2011, at the sites of FD1NPP (main gate, a north point of administration building, and west gate) and FD2NPP (MP-1) located approximately 10 km south of the FD1NPP once and twice per day, respectively. Atmospheric sampling was performed with monitoring cars, and the measurements of radionuclides collected on the first and second filters were made by a Ge semiconductor detector at FD2NPP. FEPC^14^ also started to measure the atmospheric ^131^I concentrations on March 17, 2011, at six sites in the eastern Fukushima prefecture (Fig. 6), although the ^137^Cs concentrations were not available. We also used these data to compare with the data by MEXT and DOE/NNSA.

The sampling equipment used for the first filters (HE-40T or HE-40TA: glass fiber filter) and the second filters (CHC-50: charcoal cartridge filter impregnated with 10% TEDA) was based on the reference by MEXT^44^. For the first filter, however, the HE-40TA filters were used only at NSRI^15^ and two monitoring sites (MP2 and MP6) of ORDC.^17^ DOE/NNSA also used glass fiber filters and charcoal cartridge filters impregnated with 5% TEDA to collect aerosols and volatiles, respectively^45^, while the sampling equipment was made in the U.S.A. It is noted that METI, MEXT, and JCAC (except for three samples) measured only the combined total radionuclides of the first and second filters. For our analysis on the ratio of ^131^I(a)* to the total ^131^I(a + g)*, the data from NSRI and JCAC (for three samples) were used. For the data by DOE/NNSA, however, we only used the total ^131^I(a + g) concentrations at this stage because aerosol ^131^I(a) and gaseous ^131^I(g) concentrations by DOE/NNSA were not consistent with those by NSRI. We only analysed ^131^I(a + g) data higher than approximately 100 Bq m^−3^ among the many data measured by MEXT and DOE/NNSA, because our purpose was to categorize the ratios of ^131^I(a + g)*/^137^Cs for major plumes.

Environmental contamination of the measurement/sampling system was reported by NSRI^15^ in Tokai and by CTBTO^22^ in Takasaki due to the effect of radioactive plumes with high radionuclides concentrations. At NSRI, the background counts were subtracted from the sample measurements after March 16, 2011, because the measurement system was suspected to be contaminated by radioactive plumes from FD1NPP^15^. Furthermore, Ohkura *et al*.^46^ performed additional experiments to re-evaluate the original data. At CTBTO^22,47^ in Takasaki, because the measurement system was contaminated after the plume from the FD1NPP on March 15, they corrected the data of ^134^Cs and ^137^Cs by subtracting the background counts from the original data after carrying out the blank test.

The surface meteorological data were used in the Automated Meteorological Data Acquisition System (AMeDAS) network by the Japan Meteorological Agency (JMA)^34^. The maps of precipitation amount analysed every 30 minutes by radar-AMeDAS by the JMA^42^ were also used. In addition, maps of wind distribution at a height of 1000 hPa every three hours in March 2011 were used, which were calculated with mesoscale objective analysis by the JMA^48^.

### Possible reasons why the radiocaesium concentrations were measured on the second filters

According to the radionuclide data measured at NSRI^15^, the radiocaesium concentrations were observed not only on the first filter but also on most of the second filter (CHC-50). Lebel *et al*.^28^ emphasized that the ^137^Cs concentrations on the second filter at NSRI were caused by the radiocaesium into the second filter through the first filter because the NSRI used the HE-40T filter for the first filter, with a minimum collection efficiency of approximately 50% for 0.1 µm droplets of DOP particles by Kinouchi *et al*.^49^. Therefore, Lebel *et al*.^28^ used corrected data for gaseous ^131^I(g) and particulate ^131^I(a) by calculating the penetration rates of ^137^Cs through the first filter.

**Figure 8:**
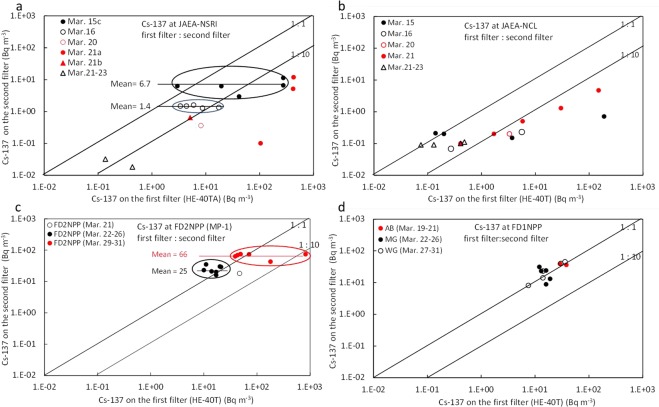
Scatter diagram between the ^137^Cs concentrations on the first filters and the ^137^Cs concentrations on the second filters. (**a)** Relationship at NSRI^15^ using the HE-40TA filter for the first filter. Numerical values 6.7 and 1.4 in the mean represent the averaged ^137^Cs concentrations on the second filters on March 15c and on March 16, respectively. **(b**) Relationship at NCL^16^ using the HE-40T filter for the first filter. (**c**) Relationship at the monitoring post (MP-1) of FD2NPP^13^ using the HE-40T filter for the first filter. Numerical values 25 and 66 in the mean represent the averaged ^137^Cs concentrations on the second filters on March 22–26, and March 29–31, respectively. (**d**) Relationship at the monitoring points of FD1NPP^13^ using the HE-40T filter for the first filter.

However, after the investigation of all ^137^Cs data on the first and second filters measured at all sites, we determined to use only the ^137^Cs concentrations collected on the first filter and have not used those collected on the second filter for the following reasons. (1) In the measurements at NSRI, Ohkura *et al*.^15^ did not use the HE-40T filters but actually used the HE-40TA filters for the first filter. The HE-40TA filter was developed to improve the collection efficiency of aerosol radioiodine in the HE-40T filter. Kinouchi *et al*.^49^ also showed that the collection efficiency of the HE-40TA filter was approximately 90% within a particle diameter range of 0.07–0.1 µm. This strongly suggests that even small particles with diameters of approximately or less than 0.1 µm can be collected on the first HE-40TA filter. (2) Before a high radiation dose rate was observed at NSRI at 5:00 (JST) on March 15, 2011, all ^137^Cs concentrations on the second filter were below the detection limits even when a higher ^137^Cs concentration of 161 Bq m^−3^ was measured at approximately 4:30 (from 4:25 to 4:45 JST; Mar. 15b in Figs 2 and 4a). This indicates no contamination at that time. (3) We analysed the ^137^Cs data at NSRI^15^, and found no relationship for the ^137^Cs concentrations between the first filter and second filter especially on Mar. 15c and Mar. 16, as shown in Fig. 8a. The ^137^Cs concentrations on the second filter were almost constant and less than 10 Bq m^−3^, while the ^137^Cs concentrations on the first filter varied within a range of one or two orders of magnitude (e.g., from 2.97 Bq m^−3^ to 366 Bq m^−3^ on March 15). We estimated that this would be caused by any contamination in the measurement/sampling system. (4) In contrast, gaseous and aerosol radionuclides were also separately measured at NCL, which is located close to NSRI, but the HE-40T filters were used as the first filter^16^. A positive relationship for the ^137^Cs concentrations was found between the first filter (HE-40T) and the second filter (CHC-50), as shown in Fig. 8b. The ^137^Cs concentrations on the second filter were estimated to be less than 1% of those on the first filter within a range of 3.0–190 Bq m^−3^. This means that most of the ^137^Cs particles collected on the second filter were caused by the penetration of smaller particles approximately or less than 0.1 µm in diameter through the first filter (HE-40T). According to the low ^137^Cs concentrations of approximately 0.1 Bq m^−3^ on both the first and second filters, any contamination might happen. Hence, we did not use the ^137^Cs concentrations on the second filter even in the measurements using the first filter of HE-40T, because our purpose focuses on plumes with high radionuclide concentrations. (5) Ohkura *et al*.^46^ re-examined by additional experiments on the cause of the existence of radiocaesium on the second filter of CHC-50. They concluded that the main reason was not due to penetration of radiocaesium through the first HE-40TA filter but due to environmental contamination in the measurement/sampling system caused by the effect of plumes with high radionuclide concentrations. They also estimated that the penetration rate would be less than 5% if it had actually happened. (6) At the Oarai Research and Development Center (ORDC), which is located approximately 20 km south of NSRI, Yamada *et al*.^17^ also measured atmospheric radionuclides by using the HE-40T and HE-40TA filters for the first filter at the monitoring station (MS3) and the monitoring posts (MP2 and MP6), respectively. And a series of CP-20 and CHC-50 (10% of TEDA was impregnated) for the second filter were set to collect volatile radionuclides after the first filter at both sites. At MS3, the ^137^Cs concentrations from 6:00 to 9:00 (JST) on March 21 were 38 and 5.9 Bq m^−3^ on the first filter (HE-40T) and second filters, respectively. In contrast, At MP2, the ^137^Cs concentrations from 4:08 on March 15 to 9:00 (JST) on March 21 were 7.4 and 0.0040 Bq m^−3^ on the first filter (HE-40TA) and second filters, respectively, although the sampling duration was much longer than that at MS3. This result also demonstrates that the penetration of radionuclides through the HE-40TA filter was scarcely found when the HE-40TA filter was used as the first filter compared to use the HE-40T filter. (7) Furthermore, we also analysed the data measured on the sites of the FD1NPP and FD2NPP by TEPCO^13^, in which the first filter of HE-40T and the second filter of CHC-50 were used. At the site of FD2NPP, the averages of the almost constant ^137^Cs concentrations on the second filter were 25 and 66 Bq m^−3^ during March 22–26 and March 29–31, respectively, while the ^137^Cs concentrations on the first filter changed one or two orders of magnitude (Fig. 8c). This result also suggests that there were no relationships in the ^137^Cs concentrations between the first and second filters, and which was similar to the results at NSRI. At the monitoring post of the FD2NPP, very high radiation dose rates of 90–180 μSv h^−1^ were measured on the early morning of March 15, 2011^35^, when the polluted air masses were transported to the south from the FD1NPP. As a result, environmental contamination in the measurement/sampling system of the FD2NPP would occur, which was similar to that at NSRI. At the sites of FD1NPP, there were no relationships in the ^137^Cs concentrations between the first filter and second filter during March 19–26 (Fig. 8d). (8) The JCAC also separately measured atmospheric radionuclides collected on the first filter (HE-40T) and second filters (CHC-50) for only 3 samples, although for the other samples, radionuclides were measured together^18^. The ratios of ^137^Cs concentration on the second filter to the sum of the ^137^Cs concentrations (on the first and second filters) were 0.016, 0.025, and 0.066 on March 15–16, March 20–21, and March 22–23, 2011, respectively. The result also indicates that, even if penetration would occur, the penetration rates would be very low and less than 7% of the total ^137^Cs concentration. (9) We also checked the data by DOE/NNSA who used a similar sampling system^45^ to that in Japan, and found that most of the ^137^Cs concentrations on the second filter were less than 5% of the ^137^Cs concentrations on the first filter except for some data.

By considering all of these results, we concluded to use only the ^137^Cs concentrations measured on the first filters, and did not use them on the second filters for all data separately measured at NSRI, NCL, FD1NPP, FD2NPP, JCAC, and DOE/NNSA. In the case of ^131^I, part of the ^131^I concentrations measured on the second filters was likely to be contaminated as was ^137^Cs. However, the contamination of ^131^I on the second filters at NSRI was estimated to be at most approximately 100 Bq m^−3^, according to the ^137^Cs contamination of approximately 10 Bq m^−3^ at NSRI on March 15. Accordingly, the uncertainty of the ^131^I concentration is estimated to be, at most, less than 10% of the measured ^131^I at NSRI. Hence, we used both the gaseous ^131^I(g) and particulate ^131^I(a) concentrations measured on the first and second filters, respectively, without any corrections, although Lebel *et al*.^28^ corrected the ^131^I data by calculating the penetration efficiency of ^137^Cs.

## Supplementary information

Supplementary Information

## Data Availability

The data which the authors analysed are available from the references or from the corresponding author upon reasonable request.
