# Supplementary material for: Dynamics of atmospheric 131I in radioactive plumes in eastern Japan immediately after the Fukushima accident by analysing published data
Source: Sci Rep. 2019 Sep 13;9:13240. doi: 10.1038/s41598-019-49379-4 (PMC6744483; doi:10.1038/s41598-019-49379-4)
Supplement: Supplementary file 1 — Supplementary Information [file 41598_2019_49379_MOESM1_ESM.pdf]

Supplementary information

Dynamics of atmospheric ^{131}I in radioactive plumes in eastern Japan immediately after the Fukushima accident by analysing published data

Haruo Tsuruta[1*], Yuichi Moriguchi[2], & Teruyuki Nakajima[3]

[1] Remote Sensing Technology Center of Japan, Minato-ku, Tokyo 105-0001, Japan
[2] Graduate School of Engineering, The University of Tokyo, Bunkyo-ku, Tokyo 113-8656, Japan
[3] Earth Observation Research Center, Japan Aerospace Exploration Agency, Sengen, Tsukuba 305-8505, Japan

Corresponding author
*Haruo Tsuruta
Tel: +81-3-6435-6784, Fax: +81-3-5777-1581
E-Mail: tsuruta@env.t.u-tokyo.ac.jp

Table S1 Days and areas of transport of plumes/polluted air masses in the Fukushima and Kantou areas during March12-25, 2011[8].

Plume	Day of March, 2011														Range of plumes				Source	Ref.
No.	12	13	14	15	16	17	18	19	20	21	22	23	24	25	Hamadori		Nakadori	Kantou	Unit	
															North	South				
p1	○														○				1	8
P1v	●														●				1	8
P1	●	→													●				1	5
P1'		●													●				1	8
p2				○												○		○	1 and/or 3	7
P2				●												●		●	2 and/or 3	5
P3				●	→										●̲		●̲		2 and/or 3	5
P4					●											●̲		●	2 and/or 3	5
P4'					●											●			2 and/or 3	8
P5							●								●				2 and/or 3	5
P5'							●									○			2 and/or 3	8
P6								●							●				2 and/or 3	5
P7									○									○	2 and/or 3	5
P8									●	→					●		●		2 and/or 3	5
P8'									●							●			2 and/or 3	8
P9										●					○	●̲		●̲	2 and/or 3	5
P9'										●						●			2 and/or 3	8
P10													●		●				1	8
P11														●	●				2 and/or 3	8

No.: Number of major plumes transport from FD1NPP in the early period after the accident.
Hamadori, Nakadori, and Kantou: The location of these areas is shown in Fig. S2.
● : Maximum ^{137}Cs concentrations ≥ 100 Bq m^{-3}.
○ : Maximum ^{137}Cs concentrations < 100 Bq m^{-3}.
→: High ^{137}Cs concentrations continued to the following morning.
● : Precipitation was observed in the area.

Table S2 Measurement periods and points of the data in March 2011 analysed in this study. Measurement points are shown in Figs. 1, 6, and S5

Institute/Agency	Date	No.*	Measurement periods (JST) and [measurement points]
METI	Mar. 12	3	8:37-8:47 [b], 8:39-8:49 [a], 13:20-13:35 [e]
MEXT	Mar. 20	6	12:40-13:00 [24], 13:57-14:17 [25], 14:13-14:30 [5], 14:13-14:33 [4], 14:15-14:35 [15], 18:30-18:50 [1]
	Mar. 21	3	13:00-13:40 [6], 13:50-14:32 [6], 14:43-15:24 [6]
	Mar. 22	2	14:55-15:34 [6], 15:50-16:30 [6]
	Mar. 23	2	13:15-13:58 [6], 14:30-15:10 [6]
	Mar. 24	1	14:55-15:15 [11]
	Mar. 25	4	13:28-13:50 [13], 14:28-14:50 [13], 15:28-15:49 [13]
DOE/NNSA	Mar. 22	4	5:28- [D], 6:01- [E], 6:33- [F], 7:04- [G]
FD1NPP	Mar. 19-21	3	11:53-12:13 (Mar. 19), 1:41-2:01 (Mar. 20), 10:19-10:39 (Mar. 21)
	Mar. 22-26	5	1:10-1:30 (Mar. 22), 2:01-2:21 (Mar. 23), 5:27-5:47 (Mar. 24), 2:01-2:27 (Mar. 25), 2:00-2:20 (Mar. 26)
	Mar. 27-31	5	2:00-2:20 (Mar. 27), 2:00-2:20 (Mar. 28), 2:22-2:42 (Mar. 29), 2:00-2:20 (Mar. 30), 2:00-2:20 (Mar. 31)
FD2NPP	Mar. 19-21	6	9:15-9:25 (Mar. 19), 18:18-18:28 (Mar. 19), 11:27-11:37 (Mar. 20), 17:10-17:20 (Mar. 20), 10:40-10:50 (Mar. 21), 18:11-18:19 (Mar. 21)
	Mar. 22-26	10	10:02-10:10 (Mar. 22), 16:43-16:51 (Mar. 22), 9:40-9:48 (Mar. 23), 16:06-16:14 (Mar. 23), 9:47-9:55 (Mar. 24), 17:46-17:54 (Mar. 24), 9:41-9:48 (Mar. 25), 17:32-17:40 (Mar. 25), 10:50-10:59 (Mar. 26), 16:22-16:29 (Mar. 26)
	Mar. 27-31	10	10:52-11:00 (Mar. 27), 17:02-17:10 (Mar. 27), 10:46-10:54 (Mar. 28), 17:04-17:12 (Mar. 28), 9:51-9:59 (Mar. 29), 15:56-16:04 (Mar. 29), 9:27-9:35 (Mar. 30), 18:30-18:38 (Mar. 30), 10:07-10:15 (Mar. 31), 14:45-14:53 (Mar. 31)
FEPC	Mar. 20	5	11:25-11:37 [41], 11:14-11:32 [42], 11:08-11:26 [43]. 11:20-11:30 [46], 14:45-14:55 [46]
	Mar. 21	3	10:50-11:08 [44], 14:40-14:53 [44], 10:48-11:10 [45]
NSRI	Mar. 15a	2	1:25-1:45, 1:55-2:15
	Mar. 15b	3	3:55-4:15, 4:25-4:45, 4:55-5:15
	Mar. 15c	6	5:55-6:15, 6:25-6:45, 6:55-7:15, 7:25-7:45, 7:55-8:15, 8:25-8:45
	Mar. 16	5	6:05-6:25, 6:35-6:55, 7:05-7:25, 7:35-7:55, 8:35-8:55
	Mar. 20	3	10:35-10:55, 11:35-11:55, 12:35-12:55
	Mar. 21a	4	3:45-4:05, 4:45-5:05, 5:45-6:05, 6:45-7:05
	Mar. 21b	2	11:15-12:00, 12:10-21:00
	Mar. 21-23	4	21:10 (Mar. 21)-9:00 (Mar. 22), 9:10-21:00 (Mar. 22), 21:10 (Mar. 22)-9:00 (Mar. 23), 9:10-21:00 (Mar. 23)

NCL	Mar. 15	4	6:00-9:07, 9:07-:15:22, 15:22-20:57, 20:57-2:52 (Mar. 16)
	Mar. 16	2	2:52-9:00, 9:00-20:56
	Mar. 20	1	8:57-20:58
	Mar. 21	5	20:58 (Mar. 20)-5:56, 5:56-8:55, 8:55-12:00, 12:00-21:10, 21:10-9:00 (Mar. 22)
	Mar. 21-23	4	21:10 (Mar. 21)-9:00 (Mar. 22), 9:00-21:00 (Mar. 22), 21:00 (Mar. 22)-9:00 (Mar. 23), 9:00-21:00 (Mar. 23)
ORDC	Mar. 15-21	2	4:08 (Mar. 15)-9:00 (Mar. 21), 6:00 (Mar. 21)-9:00 (Mar. 21)
JCAC	Mar. 15-16	1	6:25 (Mar. 15)-10:00 (Mar. 16)
	Mar. 20-21	1	10:05 (Mar. 20)-11:40 (Mar. 21)
	Mar. 22-23	1	9:55 (Mar. 22)-9:25 (Mar. 23)
TIRI	Mar. 15-16	18	0:00-7:12 (Mar. 15) -- 7:00-8:00 (Mar. 16)
	Mar. 20-21	11	16:00-24:00 (Mar. 20) -- 22:00-24:00 (Mar. 22)
	Mar. 22-23	9	12:00-14:00 (Mar. 22) -- 4:00-6:00 (Mar. 23)

*: No. means the numbers of air sampling data analyzed in this study.

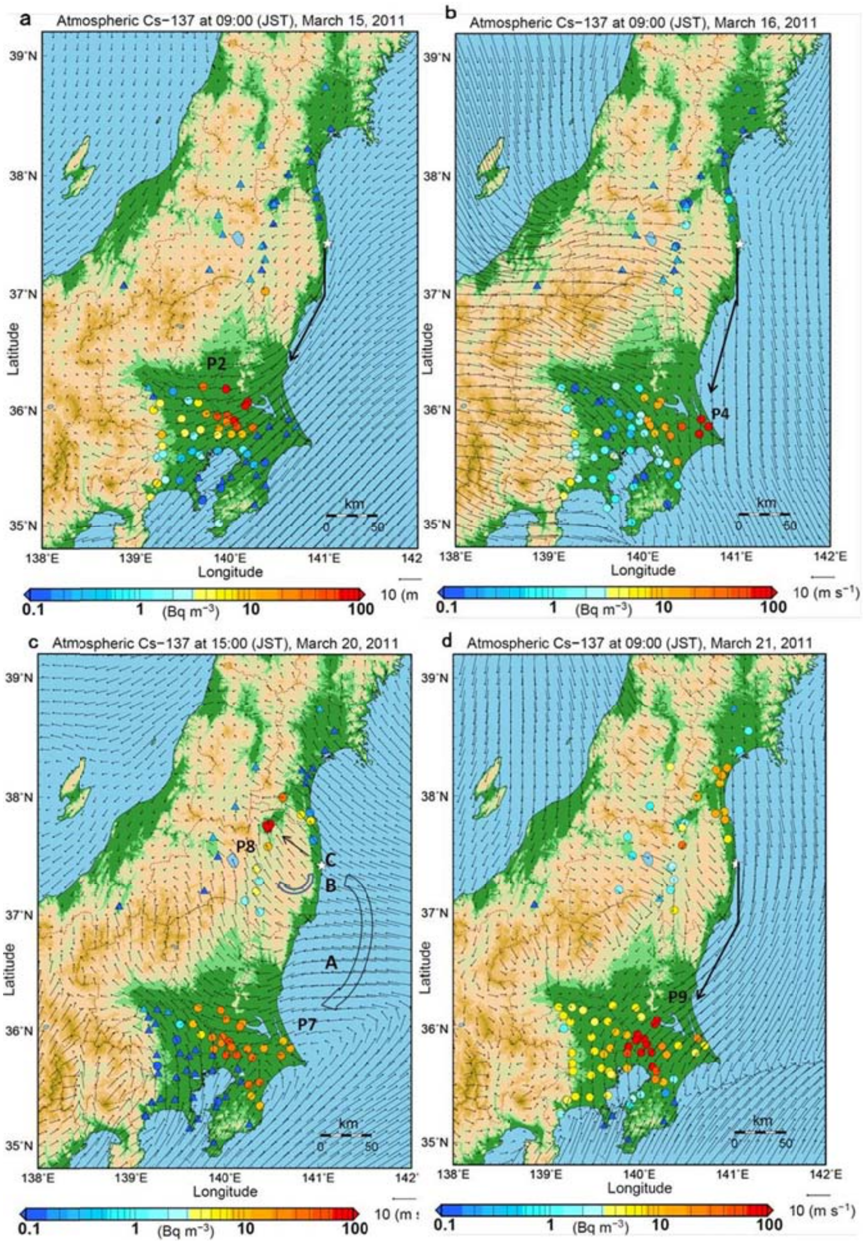

Fig. S1 Spatial distributions of atmospheric ^{137}Cs concentrations (colored dot) in major plumes and wind vectors (black arrows) at 1000 hPa calculated with mesoscale objective analysis by JMA[48] in the Kantou and southern Tohoku areas[7]. Black straight and curved lines are the schematic routes of plumes. (a) Plume P2 at 9:00 (JST), March 15, 2011. (b) Plume P4 at 9:00 (JST), March 16, 2011. (c) Plumes P7 and P8 at 15:00 (JST), March 20, 2011. Plumes were shifted clockwise as A, B, and C. (d) Plume P9 at 9:00 (JST), March 21, 2011. These four figures are slightly modified from the original ones reproduced by permission of the editor-in-chief of Earozoru Kenkyu.

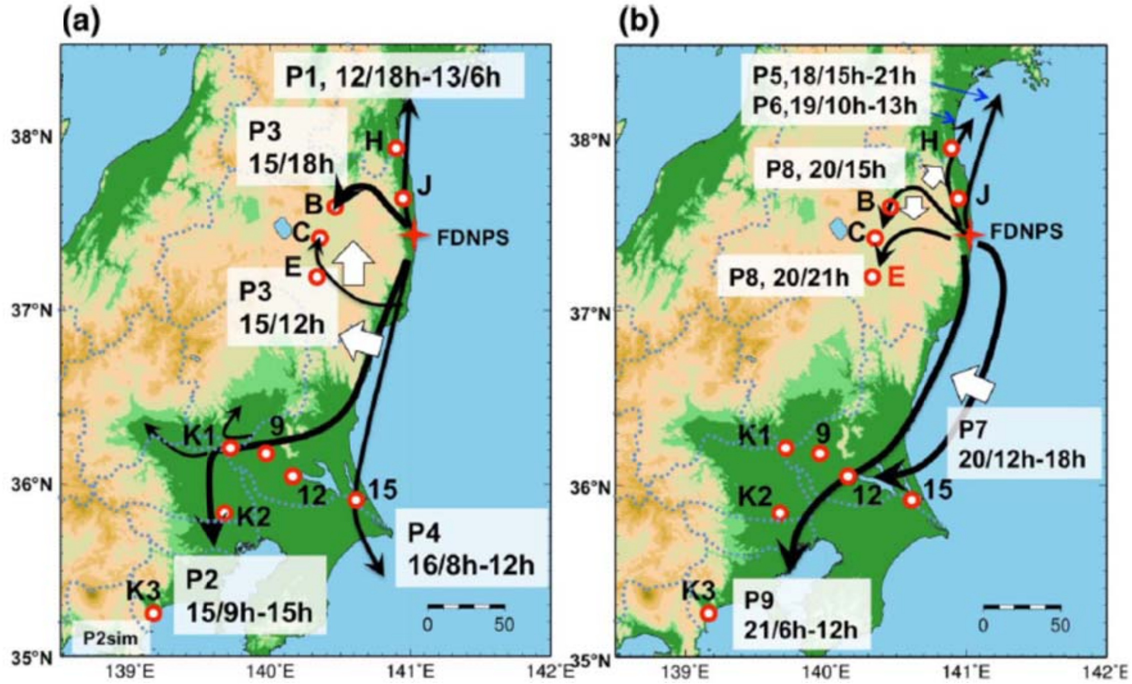

Fig. S2 Schematic diagrams of transport routes for nine plumes (P1-P9)[9]. Thick arrows indicate the general trend of the movement of each plume. The alphabetical and numerical numbers are the SPM sites. “P3, 15/18h” means Plume P3, and 18:00 (JST) on March 15, 2011. See the paper[9] for the details.

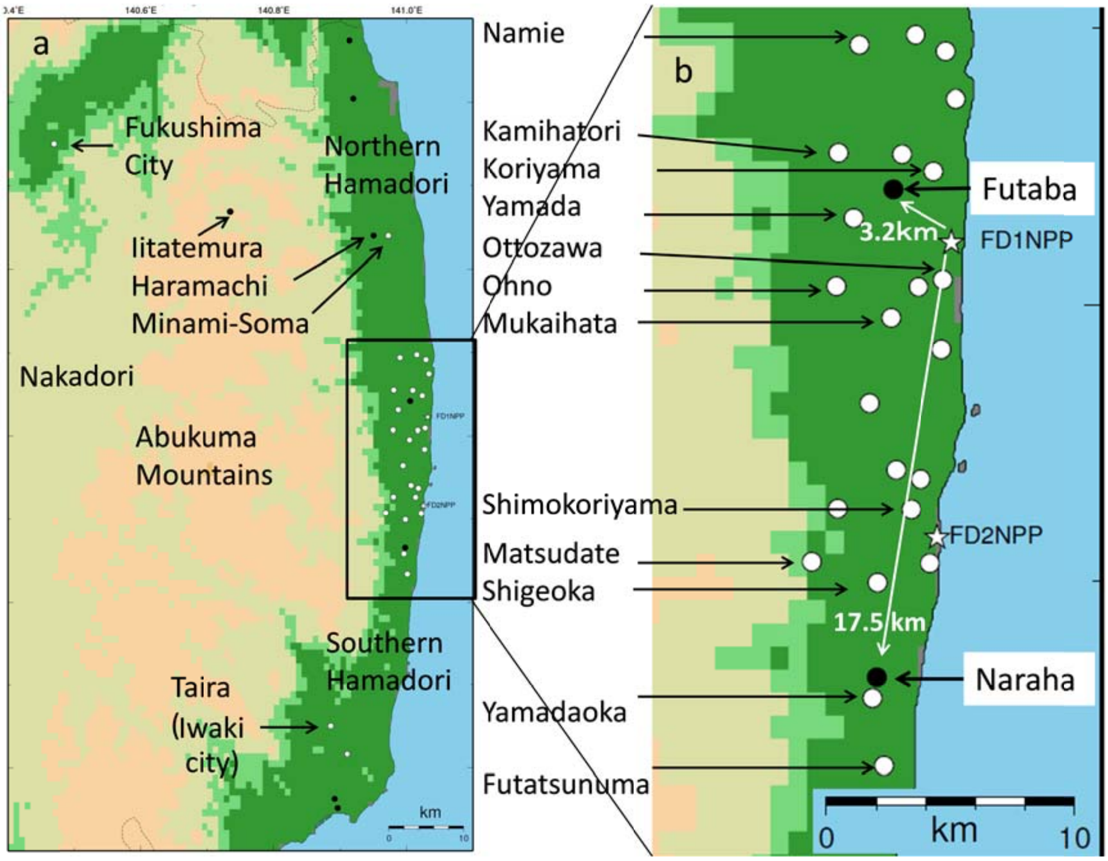

Fig. S3 Map of (a) the eastern part of Fukushima prefecture and (b) Futaba and Naraha stations for SPM concentrations (●) in the air pollution monitoring network and monitoring posts for environmental radioactivity (○) by Fukushima prefecture in Hamadori[8].

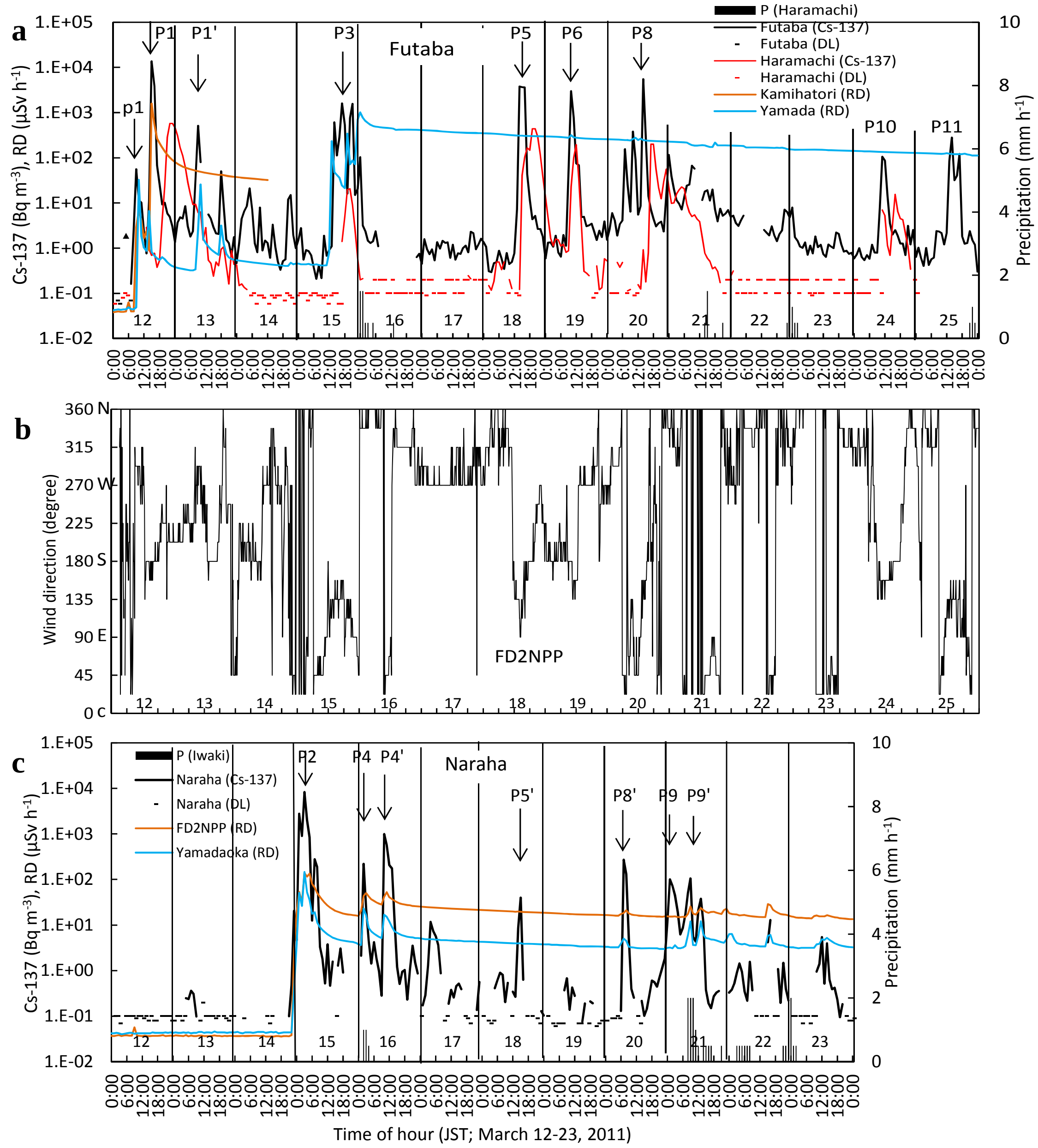

Fig. S4 Time series of hourly ^{137}Cs concentrations, radiation dose rates (RD), and wind directions during March 12-25, 2011[8]. (a) The hourly ^{137}Cs concentrations at Futaba and Haramachi, the RDs at the monitoring posts of Kamihatori and Yamada, and the precipitation at the Haramachi AMeDAS station. (b) The wind directions measured every ten minutes at the FD2NPP. (c) The hourly ^{137}Cs concentrations at Naraha, the RDs at the FD2NPP and Yamadaoka, and the precipitation at the Taira AMeDAS station in Iwaki city. Plume numbers of p1-P11 are listed in Table S1. DL means the ^{137}Cs concentration below the detection limits.

a

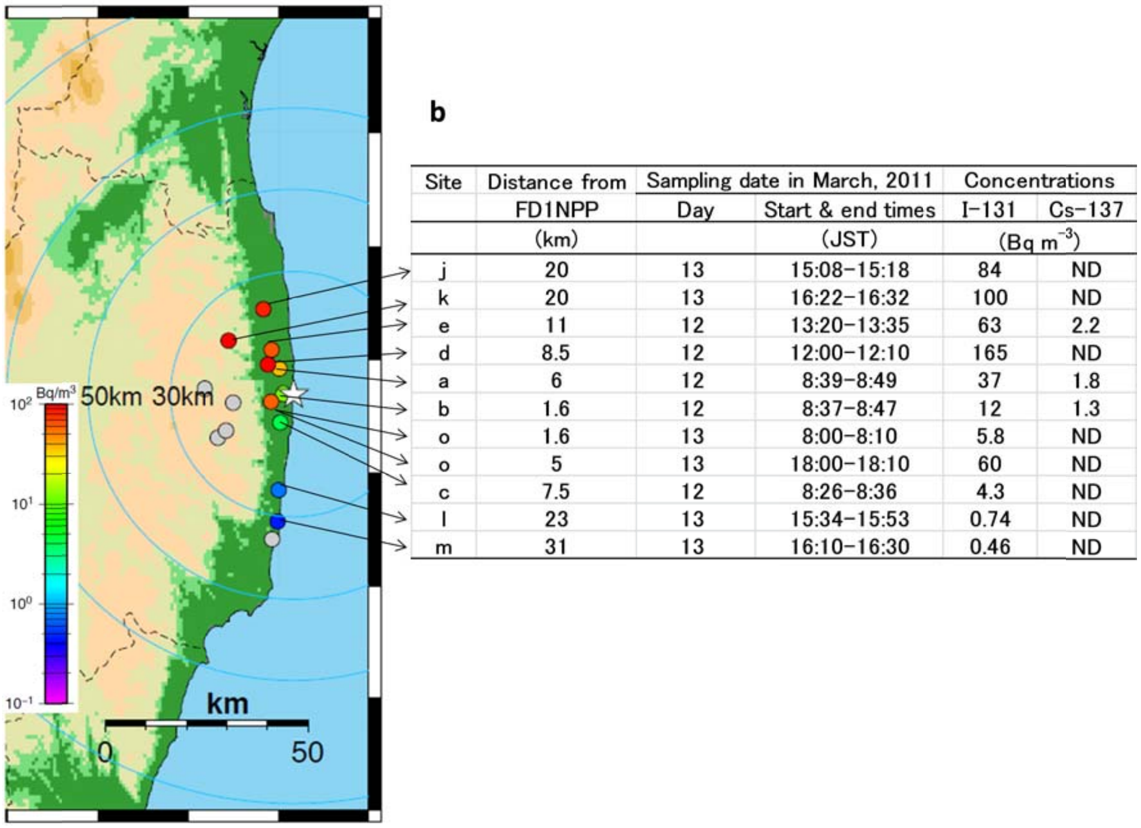

b

Site	Distance from FD1NPP (km)	Sampling date in March, 2011 Day	Start & end times (JST)	Concentrations I-131 (Bq m^{-3})	Cs-137
j	20	13	15:08–15:18	84	ND
k	20	13	16:22–16:32	100	ND
e	11	12	13:20–13:35	63	2.2
d	8.5	12	12:00–12:10	165	ND
a	6	12	8:39–8:49	37	1.8
b	1.6	12	8:37–8:47	12	1.3
o	1.6	13	8:00–8:10	5.8	ND
o	5	13	18:00–18:10	60	ND
c	7.5	12	8:26–8:36	4.3	ND
l	23	13	15:34–15:53	0.74	ND
m	31	13	16:10–16:30	0.46	ND

Fig. S5 Urgent measurements of atmospheric radionuclides by the Nuclear Emergency Response Headquarters of the Fukushima prefecture and the Ministry of Economy, Trade and Industry (METI) during March 12-13, 2011[10]. (a) Atmospheric ^{131}I(a+g) concentrations (colored dot) at temporal measurement points. The ^{131}I(a+g) concentrations with gray-colored dot were below the detection limit. (b) Properties of atmospheric radioactive measurements.

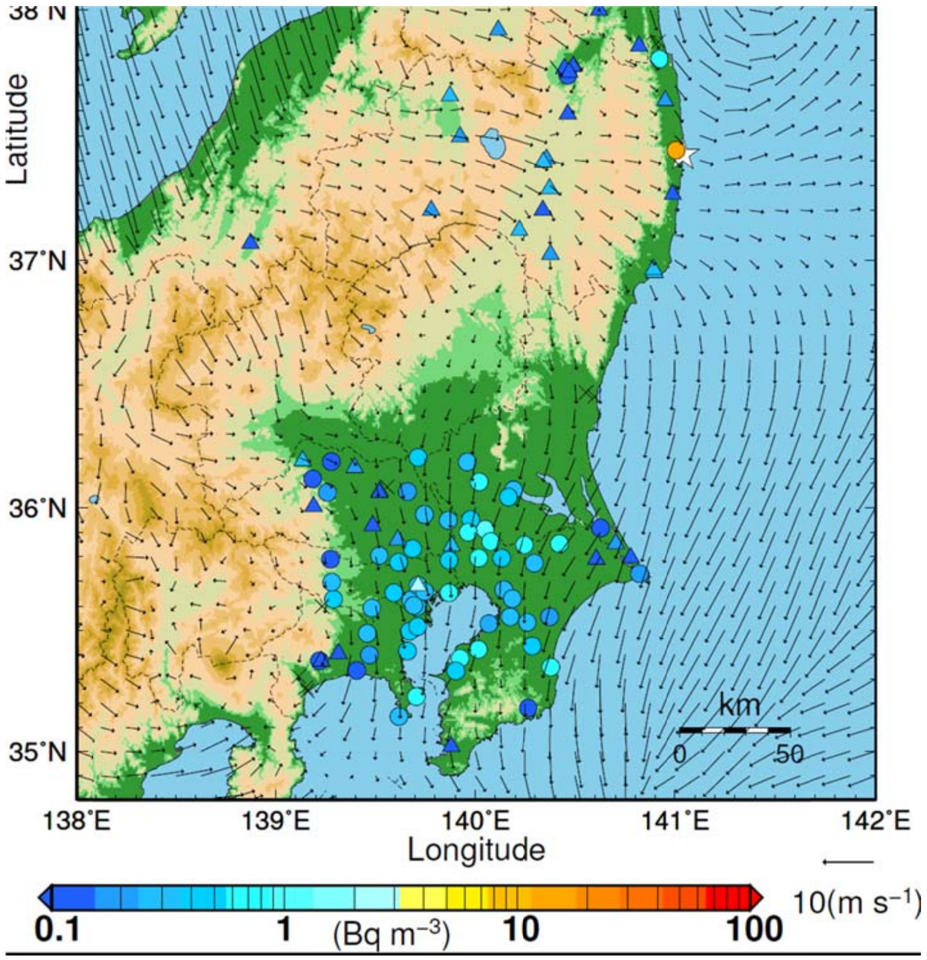

Fig. S6 Spatial distribution of atmospheric ^{137}Cs concentrations (colored dot) at the SPM stations[6] and wind vectors (black arrows) at 1000 hPa calculated with mesoscale objective analysis by the Japan Meteorological Agency[48] in the Kantou and Fukushima areas at 0:00 (JST), on March 23, 2011.

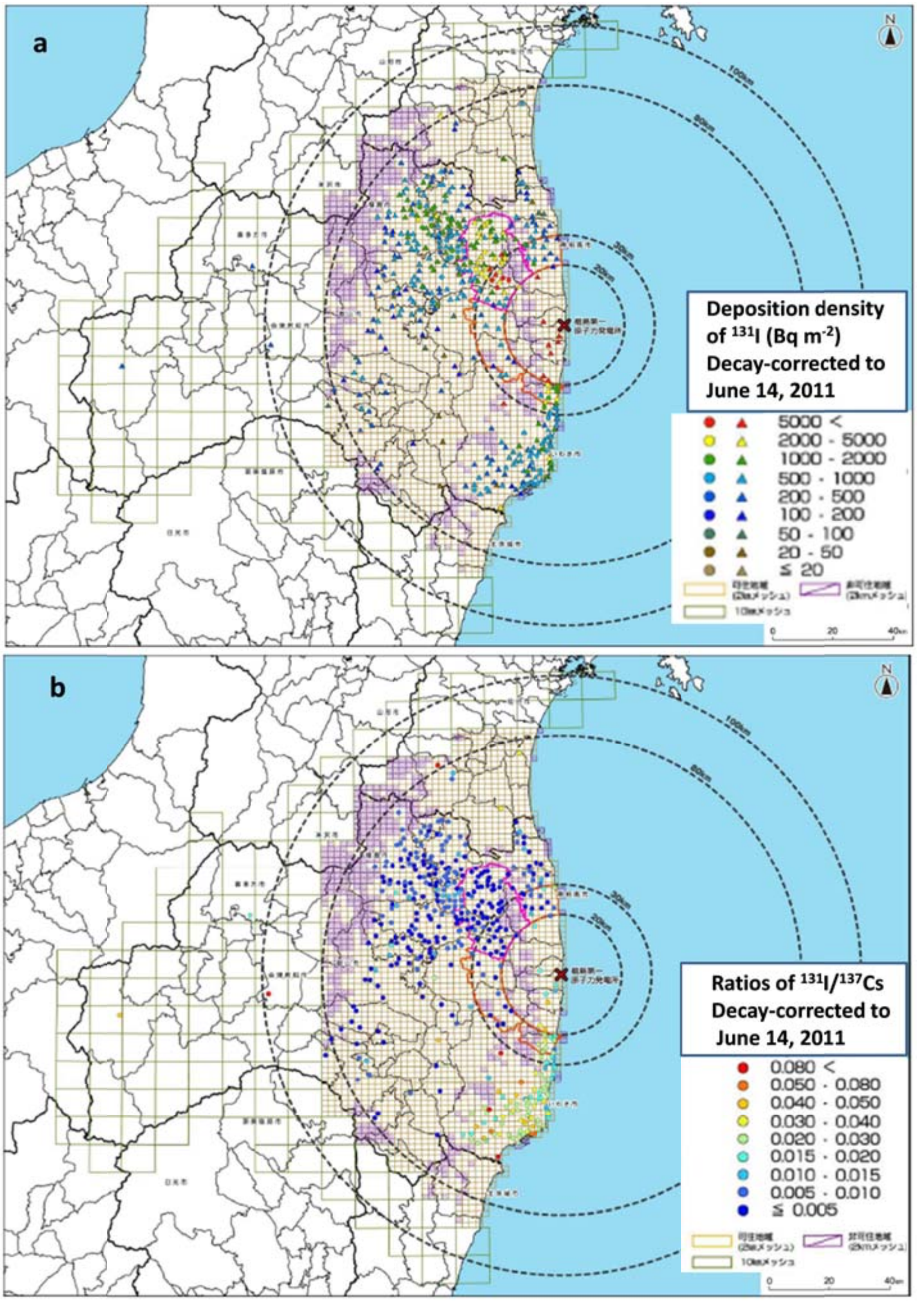

Fig. S7 In-situ measurements of ^{131}I deposition densities in the eastern Fukushima prefecture by MEXT (decay-corrected to June 14, 2011)[41]. (a) Map of ^{131}I deposition densities (Bq m^{-2}). (b) Map of the ratios of ^{131}I/^{137}Cs in deposition density.

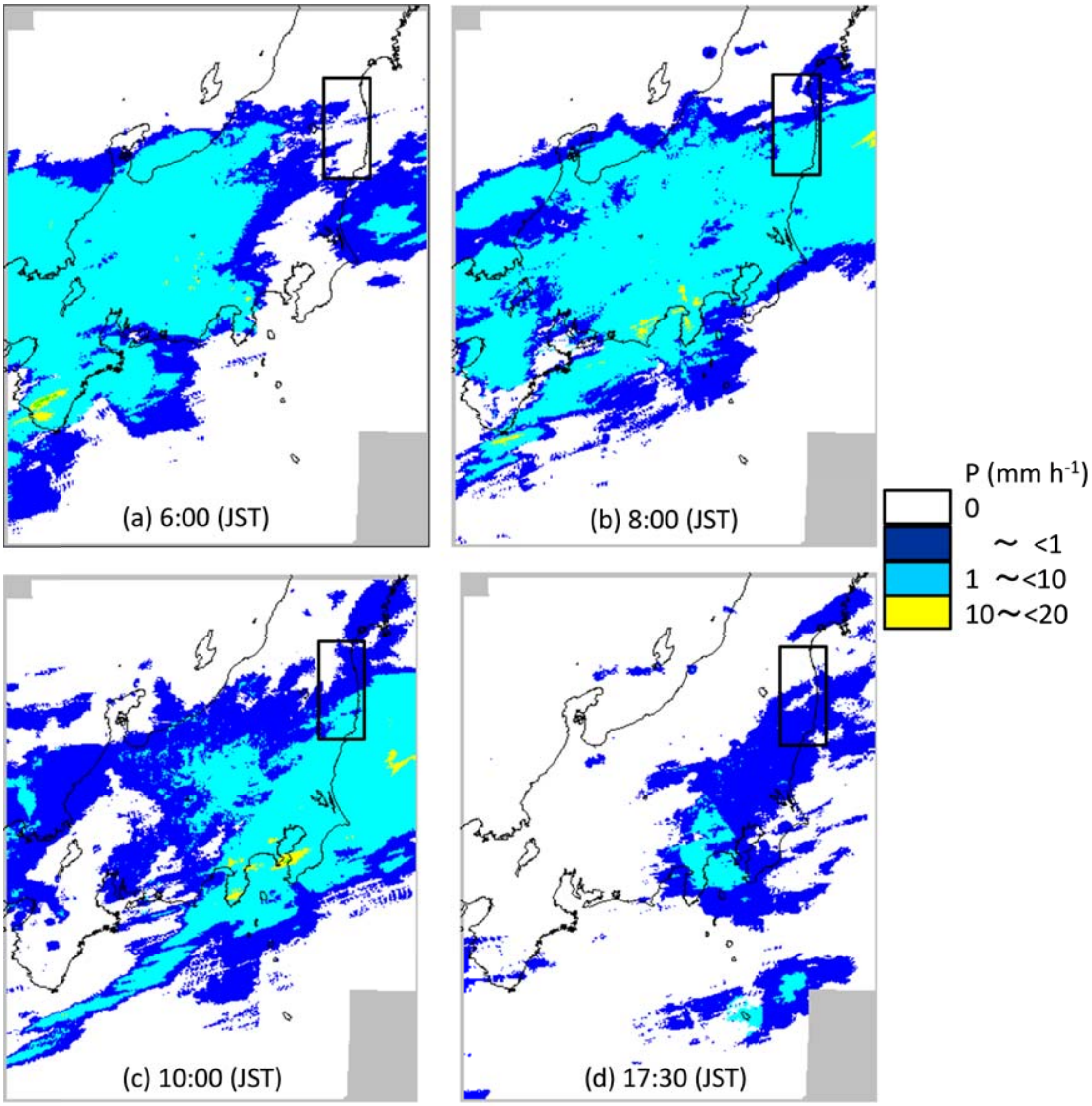

Fig. S8 Precipitation map in eastern and central Japan at (a) 6:00, (b) 8:00, (c) 10:00, and (d) 17:30 (JST) on March 21, 2011, by the analysis of radar-AMeDAS by JMA[42]. The black rectangular area is the eastern Fukushima prefecture. P (mm h^{-1}) in the color scale means the hourly precipitation amount.

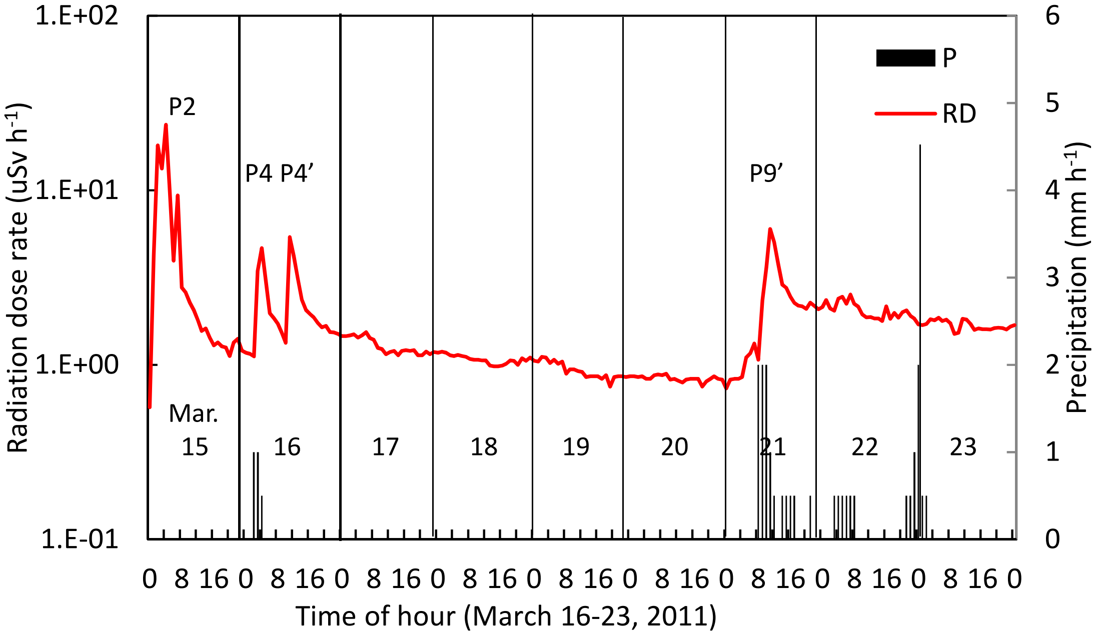

Fig. S9 Time series of radiation dose rates (RD) at Taira in Iwaki city by Fukushima prefecture[43] (Figs. 6 and S3), and the hourly precipitation (P) at the Taira AMeDAS station by JMA[34] closely located to the RD monitoring site of Taira, during March 15-23, 2011. P2, P4, P4’, and P9’ are the plume numbers which are shown in Table S1 and Fig. S4.
